# Evolutionary stochastic gradient Langevin dynamics: overcoming local traps in large-scale Bayesian sampling

**DOI:** 10.1007/s11222-026-11020-6

**Published:** 2026-11-05

**Authors:** Yushu Huang, Faming Liang

**Affiliations:** Department of Statistics, Purdue University, West Lafayette, IN 47907 USA

**Keywords:** Big Data, Evolutionary Monte Carlo, Local Trap, Population Markov Chain Monte Carlo, Snooker Operator, Stochastic Gradient Langevin Dynamics

## Abstract

The scalability of stochastic gradient MCMC algorithms has made them increasingly important for Bayesian analysis of big data. However, these algorithms are prone to getting trapped in local energy minima. We introduce a novel algorithm—evolutionary stochastic gradient Langevin dynamics (ESGLD)— that is particularly effective at mitigating local-trap behavior when simulating posterior distributions in big data scenarios. ESGLD operates with a population of Markov chains, each at a different temperature, and these chains interact through a snooker operator. The snooker operator performs a short run of SGLD along the line connecting the current sample to a selected anchor point. As long as a mode is located by other chains in the population, the target chain can transition to it by running the snooker operator, particularly when the mode is sharp and well-separated from others. The snooker operator enables the Markov chain to effectively escape local traps while maintaining invariance with respect to the target distribution. This significantly accelerates convergence, especially when the energy landscape is rugged. We provide theoretical guarantees for the convergence of the ESGLD algorithm in the big data regime and demonstrate its numerical effectiveness through simulated and real data examples, with the latter modeled by neural networks.

**Supplementary Information:**

The online version contains supplementary material available at https://doi.org/10.1007/s11222-026-11020-6.

## Introduction

After seven decades of continual development, Markov chain Monte Carlo (MCMC) has proven to be a powerful and typically unique tool for Bayesian analysis of complex data. According to their transition kernels, MCMC algorithms can be grouped into two categories, namely, Metropolis-based algorithms and Langevin-based algorithms. The former includes a large number of algorithms, such as the Metropolis-Hastings (MH) algorithm (Metropolis et al. 1953; Hastings 1970), Gibbs sampler (Geman and Geman 1984), Swendsen-Wang algorithm (Swendsen and Wang 1987), parallel tempering (Geyer 1991), evolutionary Monte Carlo (Liang and Wong 2000, 2001), stochastic approximation Monte Carlo (Liang et al. 2007), and exchange algorithms (Møller et al. 2006; Murray et al. 2006; Liang et al. 2016). These algorithms have been mainly developed to address two fundamental issues encountered in Monte Carlo simulations: local traps and intractable normalizing constants. However, they are generally inefficient in dealing with big data problems, as they require all of the data to be processed at each iteration. The mini-batch strategy is difficult to be adopted for them, as the resulting acceptance probability is generally biased.

To address the big data issue, Langevin-based algorithms have been developed, such as stochastic gradient Langevin dynamics (SGLD) (Welling and Teh 2011), stochastic gradient Hamiltonian Monte Carlo (SGHMC) (Chen et al. 2014), stochastic gradient Fisher scoring (Ahn et al. 2012), stochastic gradient Nosé-Hoover thermostats (SGNHT) (Ding et al. 2014), preconditioned SGLD (Li et al. 2016), and cyclical SGLD (Zhang et al. 2020). Refer to Ma et al. (2015) and Nemeth and Fearnhead (2019) for overviews. These algorithms can work with the mini-batch strategy, which results in an unbiased estimator for the gradient of the energy function. However, since their transitions rely solely on the gradients calculated based on the local structure of the energy landscape, these algorithms are prone to becoming trapped in local energy minima, rendering potential bias in downstream statistical inference.

To address the local trap issue, some variants of SGLD have been proposed, including replica exchange SGLD (RESGLD) (Deng et al. 2020a; Lin et al. 2022), momentum SGLD (MSGLD) (Kim et al. 2021), contour SGLD (Deng et al. 2020b), and adaptively weighted SGLD (Deng et al. 2022). However, these algorithms generally suffer from bias in simulations. Specifically, RESGLD involves parallel simulations of two Markov chains, each associated with a different temperature, and their state variables can be swapped according to the MH rule. When the mini-batch strategy is used, their energy functions become stochastic, and the acceptance probability of the swapping operator needs to be adjusted based on the variance of the stochastic energies (Ceperley and Dewing 1998). A simple plug-in of the variance estimator for the stochastic energies results in a biased acceptance rate. In the MSGLD algorithm, the drift function is intentionally biased to accelerate convergence, with the bias term being adaptively adjusted based on the gradient of past samples, ensuring the ergodicity of the simulation. Both contour SGLD and adaptively weighted SGLD are developed based on the concept of flat histogram Monte Carlo. They aim to simulate from a weighted distribution, where the importance weighting functions are adaptively learned to flatten the energy landscape of the target distribution. Unfortunately, when the mini-batch strategy is employed, the stochastic gradient of the weighted distribution is generally biased.

In this paper, we propose a new type of stochastic gradient MCMC (SGMCMC) algorithms to address the local trap issue, drawing upon the concept of evolutionary Monte Carlo (EMC) (Liang and Wong 2001). The proposed algorithm works in a population manner, where multiple chains run in parallel, each at a different temperature. Interactions between different chains occur through a snooker operator (Gilks et al. 1994; Roberts and Gilks 1994), which performs a short run of SGLD along the line connecting the current sample to a selected anchor point. As long as a mode is located by other chains in the population, the target chain can transition to it by using the snooker operator (with the mode serving as the anchor point), especially when the mode is sharp and well-separated from others. The snooker operator enables the Markov chains to effectively escape local traps while maintaining their invariance with respect to their respective target distributions. This approach significantly accelerates convergence, particularly when the energy landscape is rugged. We refer to the proposed algorithm as evolutionary SGMCMC and provide theoretical guarantees for its convergence to the target multimodal distribution in the big data regime.

The proposed algorithm differs from the EMC algorithm in two key aspects. First, the proposed algorithm implements the snooker operator using SGLD, while EMC relies on the Metropolis-Hastings algorithm. As a result, the proposed algorithm eliminates the acceptance-rejection step, ensuring that mode transitions occur without fail. The bias introduced thereby is controlled by adjusting the snooker rate, as shown in Theorem 4.4. Second, the proposed algorithm is stochastic gradient-based, making it particularly well-suited for Bayesian computation in big data scenarios. We provide in the big data regime a rigorous proof for the convergence of the proposed algorithm, which accounts for various types of bias that may be introduced by the snooker operator. See Sect. 4.2 for the detail.

The remaining part of the paper is organized as follows. Section 2 offers a concise review of the SGLD algorithm. Section 3 describes the proposed algorithm, Sect. 4 provides theoretical assurances for its convergence, and Sect. 5 demonstrates its numerical performance. Sect. 6 concludes the paper with a brief discussion.

## A brief review of stochastic gradient Langevin dynamics

Suppose that one wants to sample from a posterior distribution:

1 $$\begin{aligned} \pi (\boldsymbol{\theta }|\boldsymbol{x}_1,\ldots ,\boldsymbol{x}_N) \propto \pi _0(\boldsymbol{\theta })\prod _{i = 1}^{N}p(\boldsymbol{x}_i | \boldsymbol{\theta }), \end{aligned}$$

where $\boldsymbol{\theta }\in \mathbb {R}^d$, $\{\boldsymbol{x}_i\}_{i=1}^{N}$ represents a dataset with *N* independent samples, $\pi _0(\boldsymbol{\theta })$ is the prior distribution of $\boldsymbol{\theta }$, and p(·) is the likelihood function of the data. The sample size *N* can be very large. Suppose that both $\log \pi _0(\cdot )$ and $\log p(\cdot )$ are continuously differentiable with respect to $\boldsymbol{\theta }$, then the SGLD algorithm (Welling and Teh 2011) can be applied to simulate from a tempered posterior distribution by iterating

2 $$\begin{aligned} \hat{\boldsymbol{\theta }}_{t+1} = \hat{\boldsymbol{\theta }}_t - \eta \nabla \tilde{U}(\hat{\boldsymbol{\theta }}_t) + \sqrt{2\tau \eta }\boldsymbol{\xi }_{t+1}, \quad t=1,2,\ldots , \end{aligned}$$

where $\nabla \tilde{U}(\hat{\boldsymbol{\theta }})$ denotes an unbiased estimator of $\nabla U(\hat{\boldsymbol{\theta }})=-\nabla \log \pi (\hat{\boldsymbol{\theta }}|\boldsymbol{x}_1,\ldots ,\boldsymbol{x}_N)$ obtained with a mini-batch of data, η is the learning rate, τ is the temperature, and $\boldsymbol{\xi }_{t+1}$ denotes random noise drawn from $N(0,I_d)$. The convergence of the algorithm has been studied under different assumptions for $U(\boldsymbol{\theta })$, see e.g., Dalalyan and Karagulyan (2019) and Raginsky et al. (2017).

## Evolutionary stochastic gradient Langevin dynamics

As mentioned previously, the SGLD algorithm is prone to becoming trapped in a local energy minimum. Motivated by the success of EMC (Liang and Wong 2001) in escaping local traps, we propose the ESGLD algorithm. This algorithm operates by running a population of Markov chains in parallel, each associated with a different temperature. At each iteration, we update a portion of Markov chains by one or a few SGLD steps, while the remainder are updated using the snooker operator (defined later). The snooker operator enables the chains to escape local traps unfailingly. The ESGLD algorithm is described as follows.

Suppose that one aims to simulate from the posterior distribution (1). For simplicity, we denote $\pi (\boldsymbol{\theta }|\boldsymbol{x}_1,\ldots ,\boldsymbol{x}_N)$ by $\pi _{\tau _1}(\boldsymbol{\theta })$, where $\tau _1=1$ is the target temperature. Similarly, we define $\pi _{\tau _i}(\boldsymbol{\theta }^{(i)}) \propto (\pi (\boldsymbol{\theta }^{(i)}|\boldsymbol{x}_1,\ldots ,\boldsymbol{x}_N))^{1/\tau _i}$ for $\tau _1< \tau _2< \cdots <\tau _m$, forming a temperature ladder. Instead of simulating directly from $\pi _{\tau _1}(\boldsymbol{\theta })$, we propose simulating from the joint distribution:

3 $$\begin{aligned} \pi _{\boldsymbol{\tau }}(\boldsymbol{\theta }^{(1)}, \ldots ,\boldsymbol{\theta }^{(m)})= \prod _{i=1}^m \pi _{\tau _i}(\boldsymbol{\theta }^{(i)}), \end{aligned}$$

where $\boldsymbol{\tau }=(\tau _1,\tau _2,\ldots ,\tau _m)$, and each $\boldsymbol{\theta }^{(i)}$ represents an individual Markov chain. At each iteration of ESGLD, each individual Markov chain can undergo one of two possible moves: mutation or snooker. Suppose the state $\boldsymbol{\theta }_t^{(i)}$ of Markov chain *i* needs to be updated. If the mutation move is selected, we update $\boldsymbol{\theta }_t^{(i)}$ by running SGLD for one or a few steps. If the snooker move is selected, we update $\boldsymbol{\theta }_t^{(i)}$ using the following procedure, which aims to draw a new sample from a marginal distribution of $\pi _{\tau _i}(\boldsymbol{\theta }^{(i)})$ defined along a random line passing through $\boldsymbol{\theta }_t^{(i)}$:

(i) (*Snooker selection*) Select a snooker point from the remaining Markov chains with a probability proportional to $w_j \propto e^{-U(\boldsymbol{\theta }_t^{(j)})/t_s}$, where $0< t_s <1$ is called the selection temperature.
(ii) (*Direction sampling*) Perform SGLD sampling on the direction $\boldsymbol{e}=(\boldsymbol{\theta }_t^{(i)} - \boldsymbol{\theta }_t^{(j)})/\Vert \boldsymbol{\theta }_t^{(i)}-\boldsymbol{\theta }_t^{(j)}\Vert$ from the distribution $f(r) \propto |r|^{d - 1}\pi _{\tau _i}(\boldsymbol{\theta }_t^{(j)} + r\boldsymbol{e})$, where $\boldsymbol{\theta }_t^{(j)}$ denotes the anchor point.
(iii) (*Output*) Set $\boldsymbol{\theta }_{t+1}^{(i)}=\boldsymbol{\theta }_t^{(j)}+ r \boldsymbol{e}$, where *r* denotes the sample drawn from *f*(*r*) in step (ii).

The pseudocode for the snooker operator is given in Algorithm 1, while that for the full algorithm is given in Algorithm 2. In both algorithms, $\boldsymbol{\theta }_t^{(\cdot )}$ is replaced by $\hat{\boldsymbol{\theta }}_t^{(\cdot )}$ to reflect the approximate nature of the samples. The approximation errors will be accounted for in the convergence analysis of ESGLD, see the proof of Theorem 4.4.

**Algorithm 1:**
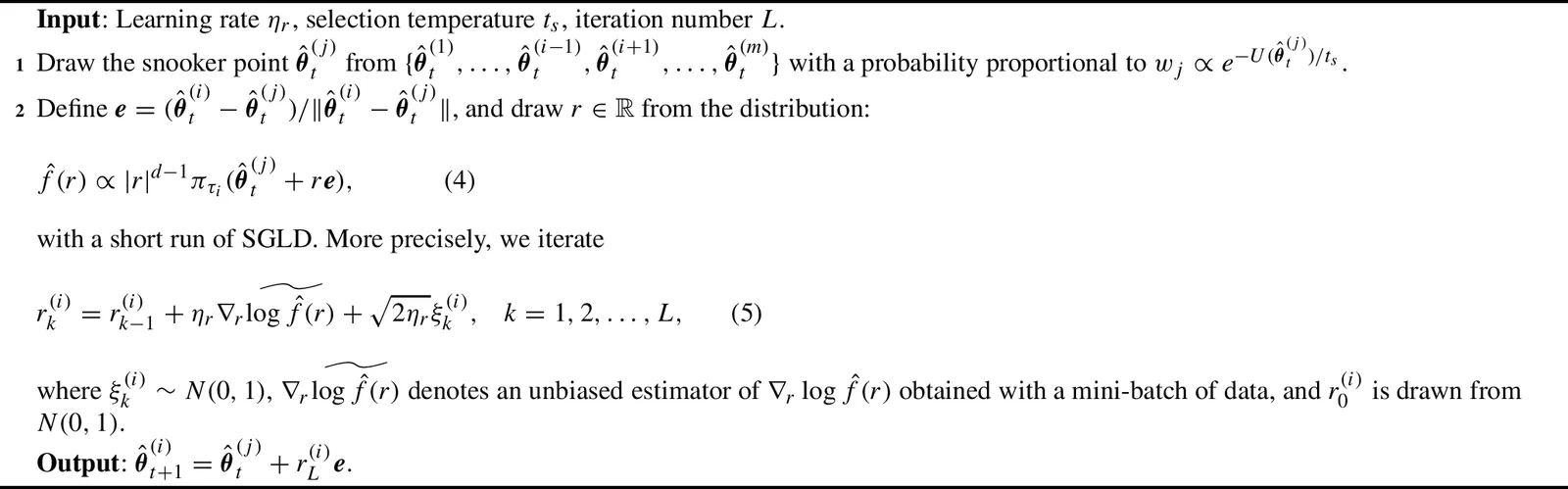
Snooker operator for chain $\hat{\boldsymbol{\theta }}_t^{(i)}$

**Algorithm 2:**
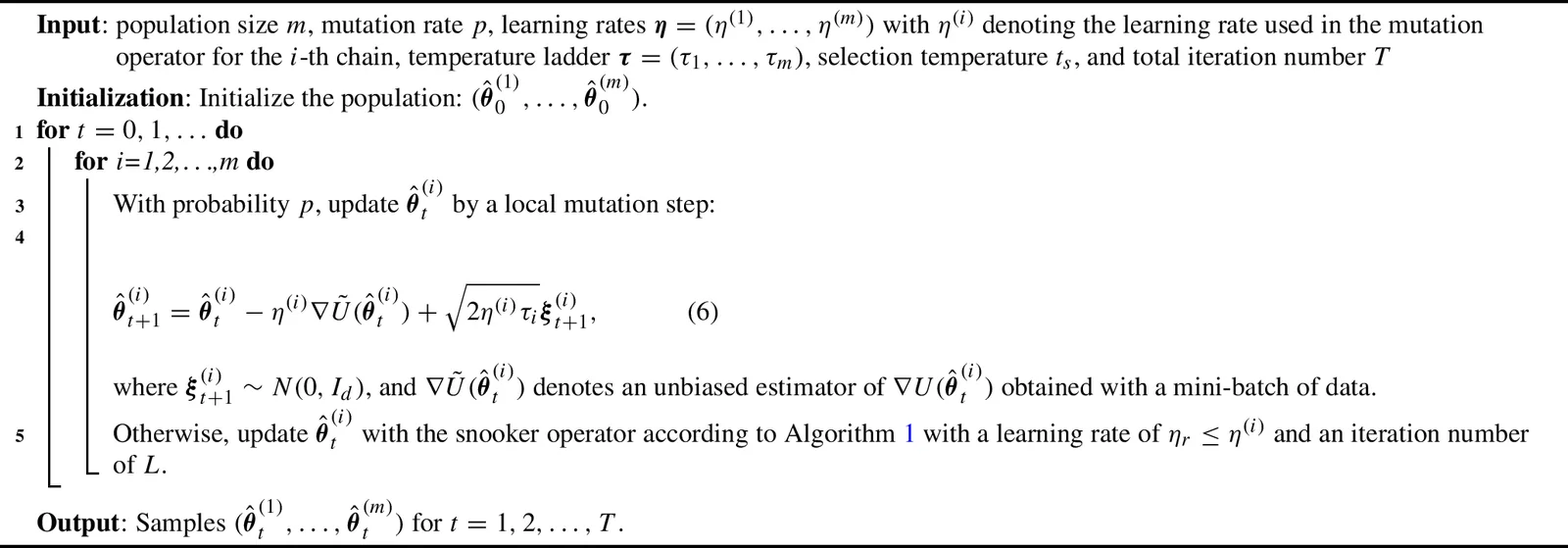
ESGLD Algorithm

Regarding the ESGLD algorithm, we have a few remarks:

### Remark 3.1

**[Snooker operator enables long-distance moves and mode transitions]** Compared with other stochastic gradient–based algorithms, such as SGLD, SGHMC, RESGLD, and contour SGLD, ESGLD can perform simulations in a dimension-reduced space via the snooker operator (i.e., along the direction $\boldsymbol{e}$). Moreover, it naturally favors long-distance moves, as reflected by the factor $|r|^{d-1}$ in the density *f*(*r*). The snooker operator is also particularly useful for mode transitions: it tends to generate samples from modes that lie along the direction $\boldsymbol{e}$ and are far from the current mode, as suggested by the two factors in the density $f(r)\propto |r|^{d-1} \pi _{\tau }(\boldsymbol{\theta }_t+r\boldsymbol{e})$.

### Remark 3.2

[**Without acceptance/rejection decisions**] Compared with EMC, ESGLD eliminates the need for acceptance/rejection decisions for the sample of *r*, allowing the snooker move to occur without fail. This ensures that as long as a mode is located by other Markov chains, the target chain can transition to it (or a similar mode along the line $\boldsymbol{e}$) using the snooker operator by selecting the mode as the snooker point. See Fig. 2 for an illustration of snooker moves. The bias introduced by the approximation error in the sample of $\hat{f}(r)$ can be controlled by selecting an appropriate snooker rate, as shown in Theorem 4.4. Additionally, ESGLD supports the use of a mini-batch strategy, making it scalable. These features make ESGLD particularly valuable for Bayesian computation in big data scenarios, especially when the energy landscape of the posterior distribution is rugged.

### Remark 3.3

**[Temperature ladder]** Concerning the temperature ladder, it is crucial to ensure that $\tau _m$ is sufficiently high to enable the hottest Markov chain to move freely, facilitating thorough exploration of the sample space. For sampling from the target posterior distribution $\pi (\boldsymbol{\theta }|\boldsymbol{x}_1,\ldots ,\boldsymbol{x}_N)$, we set $\tau _1=1$. The selection of intermediate temperatures provides more flexibility since, unlike parallel tempering (Geyer 1991), ESGLD does not entail state swapping between different chains. Lastly, it is noteworthy that if our objective is to minimize the energy function $U(\boldsymbol{\theta })$, we can set $\tau _1$ to a smaller value.

## Convergence analysis

### Continuous time convergence

Since ESGLD blends SGLD moves with snooker moves, its convergence behavior depends on both the SGLD and snooker algorithms. The continuous-time Langevin dynamic is known to possess a unique invariant probability measure, the Gibbs-Boltzmann distribution. Under suitable conditions, such as those described in Roberts and Tweedie (1996); Stramer and Tweedie (1999a, 1999b), and Mattingly et al. (2002), it converges to this distribution at an exponential rate. For the snooker operator, we give the following lemma, which is a restatement of Lemma 1 of Liu et al. (2000).

#### Lemma 4.1

[Lemma 1 of Liu et al. (2000)] Let $\boldsymbol{\theta }\in \mathbb {R}^d \sim \pi$, let $\boldsymbol{\phi }\in \mathbb {R}^d$ be an arbitrary fixed point, and let $\boldsymbol{e}= \frac{\boldsymbol{\theta }-\boldsymbol{\phi }}{\Vert \boldsymbol{\theta }-\boldsymbol{\phi }\Vert }$. Draw *r* from the distribution $f(r) \propto \vert r\vert ^{d - 1}\pi (\boldsymbol{\phi }+ r\boldsymbol{e})$, the conditional density function of $\boldsymbol{\theta }$ given $\boldsymbol{\phi }$ and $\boldsymbol{e}$, and set $\boldsymbol{\vartheta }= \boldsymbol{\phi }+ r \boldsymbol{e}$. Then $\boldsymbol{\vartheta }\sim \pi$. Additionally, if $\boldsymbol{\phi }$ is generated independently of $\boldsymbol{\theta }$, then $\boldsymbol{\vartheta }$ is independent of $\boldsymbol{\phi }$ and has density $\pi (\boldsymbol{\vartheta })$.

Note that the anchor point $\boldsymbol{\phi }$ is only required to be fixed within a given snooker move; it may vary across iterations. If $\boldsymbol{\phi }$ is random but independent of the current state being updated, the snooker output remains π-distributed. Therefore, if we blend snooker moves with a continuous-time Langevin diffusion process, the resulting stochastic process still preserves the Gibbs-Boltzmann distribution $\pi _{\boldsymbol{\tau }}(\boldsymbol{\theta }^{(1)}, \ldots , \boldsymbol{\theta }^{(m)})$ as its invariant distribution. We formally state this result in the following proposition.

#### Proposition 1

(Snooker-Langevin Diffusion) Suppose $\nabla _{\boldsymbol{\theta }} U$ $(\boldsymbol{\theta })$ is continuously differentiable and there exist some positive constants *R*, *M*, and β such that

7 $$\begin{aligned} \langle \boldsymbol{\theta }, \nabla _{\boldsymbol{\theta }} U(\boldsymbol{\theta }) \rangle \ge M \Vert \boldsymbol{\theta }\Vert ^2-\beta , \quad \Vert \boldsymbol{\theta }\Vert >R. \end{aligned}$$

Let $\{\eta ^{(i)}\}_{i=1}^m$ be *m* positive real numbers, and let *p* denote the snooker move rate at each time *t*. The population Markov chains $\{\boldsymbol{\theta }_t^{(1)}, \ldots \boldsymbol{\theta }^{(m)}_t\}$ follows the Snooker-Langevin diffusion:

1. Independent of others, each chain $\boldsymbol{\theta }^{(i)}_t$ follows the first order Langevin diffusion
  $$ d \boldsymbol{\theta }^{(i)}_t= -\nabla _{\boldsymbol{\theta }} U(\boldsymbol{\theta }^{(i)}_t)dt+ \sqrt{2 \tau _i} d \boldsymbol{W}^{(i)}_t, $$
  over the time interval $[0,\infty )\backslash \{t \eta ^{(i)}\}_{t = 1}^{\infty }$, where $\boldsymbol{W}_t^{(i)}$ denotes the Brownian motion.
2. At each of the discrete time points $\{t\eta ^{(i)}\}_{t = 1}^{\infty }$, randomly select *pm* chains to undergo the first-order Langevin diffusion independently, update the remaining (1-p)m chains by the snooker operator independently, and jump over the time period from $t\eta ^{(i)}$ to $(t+1)\eta ^{(i)}$.

Suppose that the Markov chains are subject to the Langevin diffusion only during their warm-up period. Then $\pi _{\boldsymbol{\tau }}(\boldsymbol{\theta }^{(1)},$ $\ldots ,\boldsymbol{\theta }^{(m)})$ is invariant with respect to the snooker-Langevin diffusion.

The proof of Proposition 1 is straightforward: It follows from Theorem 2.1 of Roberts and Tweedie (1996) that π is the invariant distribution for the Langevin diffusion process and, for any initial point, it can reach equilibrium as *t* becomes large. Therefore, by Lemma 4.1, blending the Langevin diffusion and snooker moves will not alter its invariant distribution after the chains have reached equilibrium. For theoretical simplicity, we assume that the Markov chains are warmed up using only Langevin diffusion and that the snooker moves are performed sequentially. In practice, we can understand ‘the Markov chains have been warmed up’ as meaning that each of them has ever reached a local mode of the respective invariant distribution $\pi _{\tau _i}(\boldsymbol{\theta })$. We note that when (1-p)m chains are updated simultaneously, the algorithm remains valid provided that the anchor samples are chosen so that they are independent of the chains undergoing the updates. For example, the anchor samples may be selected from the chains outside the set of (1-p)m chains being updated. An extended proof for Proposition 1 and a remark for justifying the validity of simultaneous snooker updates are given in the Supplement.

### Nonasymptotic analysis

This section provides a nonasymptotic analysis for the ESGLD algorithm. Let $\boldsymbol{\theta }_t$ denote an individual chain in the population $\{\boldsymbol{\theta }_t^{(1)}, \ldots ,\boldsymbol{\theta }_t^{(m)}\}$ obtained by the snooker-Langevin diffusion process. Likewise, let $\hat{\boldsymbol{\theta }}_t$ denote the sample of the corresponding chain obtained by running the ESGLD algorithm. Let τ denote the temperature of the chains $\boldsymbol{\theta }_t$ and $\hat{\boldsymbol{\theta }}_t$. Our goal is to characterize the 2-Wasserstein distance between the distribution of $\boldsymbol{\theta }_t$ and that of $\hat{\boldsymbol{\theta }}_t$. For two distributions μ and ν, the 2-Wasserstein distance is given by

$$ \mathcal {W}_2(\mu ,\nu ) = \inf _{\{\mu ,\nu \} \in \Gamma (\mu ,\nu )} \left( \mathbb {E}\Vert \boldsymbol{\theta }- \boldsymbol{\vartheta }\Vert ^2\right) ^{\frac{1}{2}}, $$

where $\Gamma (\mu ,\nu )$ denotes the set of all couplings of μ and ν, and $\boldsymbol{\theta }\sim \mu$ and $\boldsymbol{\vartheta }\sim \nu$ denote the samples drawn from μ and ν, respectively. To facilitate this theoretical study, we first define the stochastic process that generates $\hat{\boldsymbol{\theta }}_t$.

#### Definition 1

Warm up each Markov chain in the population using the SGLD sampler. During the subsequent iterations, at iteration t+1, $\hat{\boldsymbol{\theta }}_t$ is updated as follows: With probability *p*, it is updated with a SGLD move at temperature τ: $\hat{\boldsymbol{\theta }}_{t+1} = \hat{\boldsymbol{\theta }}_t - \eta \nabla \tilde{U}(\hat{\boldsymbol{\theta }}_t) + \sqrt{2\eta \tau }\boldsymbol{\xi }_{t+1}$, where η is the learning rate, $\boldsymbol{\xi }_{t+1} \sim N(0,I_d)$; and with probability (1-p), it is updated with a snooker move performed with *L* iterations of SGLD (see Algorithm 1): $\hat{\boldsymbol{\theta }}_{t+1} = \boldsymbol{\phi }+ r_L \frac{\hat{\boldsymbol{\theta }}_t-\boldsymbol{\phi }}{\Vert \hat{\boldsymbol{\theta }}_t-\boldsymbol{\phi }\Vert }$, where $\boldsymbol{\phi }$ denotes a snooker point selected in the move.

To bound the 2-Wasserstein distance between the distributions of $\hat{\boldsymbol{\theta }}_t$ and $\boldsymbol{\theta }_t$, we impose the following assumptions. Throughout the asymptotic analysis, the notation $a_N\prec b_N$ means $\lim _{N\rightarrow \infty }\frac{a_N}{b_N}=0$, referring to a sequence of big-data problems as the sample size $N\rightarrow \infty$. Accordingly, the energy function and associated quantities may depend on *N*. For notational convenience, we may suppress the subscripts in $a_N$ and $b_N$, with their dependence on *N* understood implicitly. The parameter dimension *d*, however, is assumed to be fixed in this paper.

#### Assumption 1

There exist constants M>0 and $\beta \ge 0$ such that for any $\boldsymbol{\theta }\in \mathbb {R}^d$, $\langle \boldsymbol{\theta }, \nabla U(\boldsymbol{\theta }) \rangle \ge M \Vert \boldsymbol{\theta }\Vert ^2 - \beta$ holds, where $\beta \prec M$, and the dependence of *M* and β on the sample size *N* is left implicit.

#### Assumption 2

$U(\boldsymbol{\theta })$ is $C_g$-smooth, i.e., there exists a constant $C_g >0$ such that for any $\boldsymbol{\theta }$, $\boldsymbol{\vartheta }\in \mathbb {R}^d$, $\Vert \nabla U(\boldsymbol{\theta }) - \nabla U(\boldsymbol{\vartheta })\Vert \le C_g \Vert \boldsymbol{\theta }- \boldsymbol{\vartheta }\Vert$ holds, where $\max \{0,C_g-M\}\prec M$, and the dependence of $C_g$ on the sample size *N* is left implicit.

#### Remark 4.1

Assumptions 1 and 2 are standard stability conditions ensuring that the confining drift dominates possible local nonconvexity, which is needed for the one-step $W_2$ recursion in Theorem 4.4. A convenient sufficient condition is a *Hessian sandwich* (globally or outside a compact set): there exist constants $0<m\le L<\infty$ and R≥0 such that

$$ mI \;\preceq \; \nabla ^2 U(\theta ) \;\preceq \; LI, \qquad \forall \,\theta \text { with }\Vert \theta \Vert \ge R. $$

The upper bound implies $C_g$-smoothness, i.e., $\Vert \nabla U(\theta )-\nabla U(\vartheta )\Vert \le L\Vert \theta -\vartheta \Vert$, so one may take $C_g=L$ in Assumption 2. The lower bound yields a quadratic restoring force and implies a dissipativity inequality of the form

$$ \langle \theta ,\nabla U(\theta )\rangle \;\ge \; m\Vert \theta \Vert ^2-\beta , $$

where $\beta \ge 0$ depends only on the behavior of *U* on the compact ball $\{\Vert \theta \Vert \le R\}$. Thus one may take M=m in Assumption 1. In many statistical models (e.g., targets that are approximately quadratic in the tails or strongly convex outside a compact set), the same curvature controls both *M* and $C_g$ via the smallest and largest eigenvalues of $\nabla ^2U(\theta )$, respectively; consequently $C_g$ and *M* are naturally comparable and may even coincide (e.g., for a quadratic energy $U(\theta )=\frac{\lambda }{2}\Vert \theta \Vert ^2$ we have $C_g=M=\lambda$). Under such curvature control, the condition $\max \{0,C_g-M\}\prec M$ simply requires that the excess smoothness beyond dissipativity is of smaller order than the dissipativity level. Moreover, in many statistical models, U(θ) has an additive structure (negative log-likelihood plus prior), so the magnitude of $\nabla U(\theta )$ naturally increase with *N* and $M = O(N^\alpha )$ holds for some constant α>0.

#### Assumption 3

There exist positive constants A≺M and $B \preceq M^{1/2}$ such that $|U(\boldsymbol{\theta }_0)|\le A$ and $\Vert \nabla U(\boldsymbol{\theta }_0) \Vert \le B$ at some point $\boldsymbol{\theta }_0 \in \mathbb {R}^d$. The dependence of *A* and *B* is left implicit.

#### Assumption 4

Let $u(\boldsymbol{\theta }) = \nabla \tilde{U}(\boldsymbol{\theta }) - \nabla U(\boldsymbol{\theta })$, the stochastic gradient noise. There exists a constant $\delta \in [0,1)$ such that $\mathbb {E}\Vert u(\boldsymbol{\theta })\Vert ^2 \le 2\delta (C_g^2 \Vert \boldsymbol{\theta }\Vert ^2+B^2)$ for any $\boldsymbol{\theta }\in \mathbb {R}^d$.

#### Assumption 5

[Initial probability law $\pi _0$] The probability law of the initial point $\boldsymbol{\theta }_0$ has a bounded and strictly positive density $\pi _0$ with respect to the Lebesgue measure on $\mathbb {R}^d$, and

$$ \kappa _0:= \log \int _{\mathbb {R}^d} e^{\Vert \boldsymbol{\theta }\Vert ^2} \pi _0(\boldsymbol{\theta }) d \boldsymbol{\theta }< \infty . $$

Assumptions 1–5 have often been used in studying the convergence of SGLD, see e.g. Raginsky et al. (2017). To facilitate the convergence of the algorithm, we impose mild structural conditions on M, $C_g$, B, and β. These conditions ensure a proper balance between the smoothness and dissipativity of the energy function $U(\boldsymbol{\theta })$, which is crucial for controlling the behavior of the Langevin dynamics. The condition $\max \{0, C_g - M\} \prec M$ ensures that the dissipativity remains strong relative to the local curvature of the gradient field, a key requirement for establishing contraction in Wasserstein distance and ensuring numerical stability of the discretized process. Bounding the slack term by $\beta \prec M$ guarantees that the dissipative force remains sufficiently strong even when the potential deviates from strong convexity, while the conditions A≺M and $B \preceq M^{1/2}$ naturally hold at a reference point $\boldsymbol{\theta }_0$, e.g., a local mode. Together, these conditions strike a balance between generality and control, enabling convergence guarantees under realistic growth conditions commonly satisfied in Bayesian inference for big data problems.

To study the convergence of the ESGLD sampler, we need to analyze not only the Langevin dynamics on $\mathbb {R}^d$ induced by SGLD moves, but also the Langevin dynamics on the line $\boldsymbol{e}$ induced by the snooker operator. For the latter, we define the density function

8 $$\begin{aligned} \hat{f}(r) \propto |r|^{d-1} \pi (\boldsymbol{\phi }+r \hat{\boldsymbol{e}}), \end{aligned}$$

with $\hat{\boldsymbol{e}}= \frac{\hat{\boldsymbol{\theta }}-\boldsymbol{\phi }}{\Vert \hat{\boldsymbol{\theta }}-\boldsymbol{\phi }\Vert }$, where $\hat{\boldsymbol{\theta }}$ denotes a sample generated by the sampler. Let $\hat{U}_r = -\log \hat{f}(r)$. The next two lemmas show that $\hat{U}_r$ also satisfies the smoothness and dissipativity conditions, see Supplement A.3 and A.4 for their respective proofs. Additionally, it is easy to see that the snooker operator also satisfies Assumptions 3, 4, and 5.

#### Lemma 4.2

Suppose Assumptions 1–5 hold, then $\hat{U}_r$ is $C_{\boldsymbol{\phi }}$-smooth for some constant $C_{\boldsymbol{\phi }}\ge C_g$, with dominating probability.

#### Lemma 4.3

Suppose Assumptions 1–5 hold. Then there exist a constant $\beta _{\boldsymbol{\phi }} \ge 0$ such that $r \nabla \hat{U}_r \ge M r^2 - \beta _{\boldsymbol{\phi }}$, with dominating probability.

We say a property holds *with dominating probability* if there exists an event $\mathcal {E}$ (on which the property holds) such that for every ϵ>0 one can choose the associated constants/hyperparameters so that $\mathbb {P}(\mathcal {E})\ge 1-\epsilon$ (equivalently, the event probability can be made arbitrarily close to one, as formalized in (A.6), the proof of Lemma 4.2, and the proof of Lemma 4.3). We made this type of statements for two reasons: (i) we need to bound the region of anchor points probabilistically, and (ii) we need to restrict the sample space of $\hat{f}(r)$ to intervals where $\nabla _r \hat{U}_r$ is smooth. For the former, as implied by Assumption 1, $\pi (\boldsymbol{\theta }|\boldsymbol{x}_1,\ldots ,\boldsymbol{x}_N)$ is zero-concentrated with the tail decaying like Gaussian or faster, so it follows from Algorithm 1 that the anchor points fall into a compact set with probability 1, uniformly over all iterations (see Supplement A.2). Conditioned on this, for any ε>0, we can identify positive constants *a* and $C_{\boldsymbol{\phi }}$ such that for any selected anchor point $\boldsymbol{\phi }$ and direction $\hat{\boldsymbol{e}}$, $\int _{-\infty }^{-a} \hat{f}(r) dr + \int _{a}^{\infty } \hat{f}(r) dr \ge 1-\varepsilon$ and $\hat{U}_r$ is $C_{\boldsymbol{\phi }}$-smooth and dissipative on $(-\infty ,a] \cup [a,\infty )$. Consequently, the results of Theorem 4.4 are also stated in the same manner.

#### Theorem 4.4

Suppose Assumptions 1-5 hold and $0< \eta _r \le \eta \prec \frac{\sqrt{M}}{4C_{g}^2}$.

Let $\hat{\pi }_t$ and $\pi _t$ denote the distributions of $\hat{\boldsymbol{\theta }}_t$ and $\boldsymbol{\theta }_t$, respectively. Then, with dominating probability,

9 $$\begin{aligned}&\mathcal {W}_2^2(\hat{\pi }_{t+1}, \pi _{t+1}) \le \varrho \mathcal {W}_2^2(\hat{\pi }_{t}, \pi _{t}) + 4(1-p)L^2 \eta ^2 (\tilde{c}^{\prime } \delta ^{\frac{1}{2}}\nonumber \\&+\tilde{c}^{\prime \prime }\eta ^{\frac{1}{2}})+2p(c^{\prime } + 8\tau ) \eta + 4 p c^{\prime \prime } \delta \eta ^2, \end{aligned}$$

where $\tilde{c}^{\prime }$, $\tilde{c}^{\prime \prime }$, $c^{\prime }$, and $c^{\prime \prime }$ are some constants (depending on *M*, β, $C_g$, and *B*), $\varrho =p(1-2 M\eta +8C_g^2 \eta ^2) + (1-p)(\omega +1)<1$ by setting $p> \frac{\omega }{\omega +2M \eta -8C_g^2 \eta ^2}$ for some constant ω>0, and $L=-s \log (\eta )/\eta$ for some constant *s* (see the proof for expressions of ω and *s*).

Furthermore, if we set $p=\frac{\omega }{\omega +\eta ^{\psi }M/c_0-8C_g^2 \eta ^2}$ for some ψ>1 and $c_0>1$, then $W_2^2(\hat{\pi }_{t}, \pi _{t}) \rightarrow 0$ as $t \rightarrow \infty$, $\eta \rightarrow 0$, and $N \rightarrow \infty$.

Based on the proof of Theorem 4.4, we give the following remark concerning the temperature ladder setup for the ESGLD algorithm:

#### Remark 4.2

The convergence is established for a single chain only, which has important implications for setting up the temperature ladder. Let’s classify the population of Markov chains as the target chain and high-temperature chains. The high-temperature chains are designed to locate different modes of the distribution, with the temperature ladder appropriately selected to balance exploration and exploitation within the sample space. This balance facilitates the target chain to transition between different modes of the distribution effectively. As mentioned in Remark 3.3, ESGLD avoids state swapping between different chains, offering greater flexibility in choosing the population size and temperature ladder. In essence, ESGLD can function with just two chains: one target chain and one appropriately chosen high-temperature chain. However, in this case, balancing exploration and exploitation with a single high-temperature chain becomes more challenging; additionally, the samples drawn by the high-temperature chains may need to be stored into a “snooker pool”, which will lead to a slightly different theoretical framework than that studied in Theorem 4.4. See Sect. 6 for more discussions on this issue.

The mode-transition mechanism introduced by the snooker operator may offer significant advantages over the replica exchange algorithm (Deng et al. 2020a). The latter, being Metropolis-based, suffers from biased acceptance probabilities due to the involvement of stochastic energies, and its acceptance rate can become extremely low when the dimension of $\boldsymbol{\theta }$ is high or the temperatures of the two chains proposed for swapping are disparate. In contrast, the snooker operator, which is Langevin-based, eliminates the need for acceptance-rejection steps. This ensures that a snooker update is always executed without rejection and changes the current state with probability one. Whether the new state falls into a different mode is actually a random event, whose probability depends on the relative probability mass of the modes under the one-dimensional conditional distribution *f*(*r*).

However, this transition may introduce bias into the simulation, potentially impacting its convergence. The bias can arise from two sources: the finite number of SGLD updates performed in the snooker step and the deviation of the current sample’s distribution from the target distribution. Our proof accounts for both sources and shows that the bias can be controlled by selecting an appropriate snooker rate (i.e., 1-p) and a suitable number of iterations for the snooker operator (i.e., *L*), ensuring 2-Wasserstein convergence of the Markov chain as the number of iterations becomes large and the learning rate η tends to 0.

As indicated by our proof, the snooker rate should be determined by factors such as η, *M*, $C_g$, and ω. A closer analysis reveals that ω is negatively influenced by the moments of *f*(*r*), and thus, by *M* as well. In general, a higher snooker rate can be employed when either η or *M* is large. Our experiments showed that the performance of ESGLD is quite robust to the snooker rate, see Supplement B.1.2 for the detail.

Additionally, we note that the goal of Theorem 4.4 is not to bound the mixing time of the limiting process to its stationary distribution, but rather to derive a one-step recursive inequality for the discrepancy between the practical ESGLD iterate and the corresponding ideal (continuous-time) process in $\mathcal {W}_2$. Thus, as shown by our proof (around equation (A.19)), any spectral-gap-like dependence from Raginsky et al. (2017) affects only the snooker SGLD approximation error and is placed into an *o*(1) remainder via the choice of *L*.

Let $\hat{\pi }$ and π denote, respectively, the limits of $\hat{\pi }_t$ and $\pi _t$ as $t \rightarrow \infty$. Regarding statistical inference for π, we have the following lemma.

#### Corollary 4.5

Assume that both $\hat{\pi }$ and π have finite second moments. Let $h(\boldsymbol{\theta }): \mathbb {R}^d \rightarrow \mathbb {R}$ be a continuously differentiable function on $\mathbb {R}^d$ obeying $\Vert \nabla h(\boldsymbol{\theta })\Vert \le c_1 \Vert \boldsymbol{\theta }\Vert +c_2$ for some constants $c_1>0$ and $c_2 \ge 0$. Then $\left| \mathbb {E}_{\hat{\pi }} h(\boldsymbol{\theta }) - \mathbb {E}_\pi h(\boldsymbol{\theta }) \right| \rightarrow 0$ as $\eta \rightarrow 0$ and $N\rightarrow \infty$.

The corollary provides a rigorous foundation for performing statistical inference for π using approximate posterior samples simulated from $\hat{\pi }$. The assumptions imposed on *h*, i.e., quadratic growth, are satisfied by a broad class of inferential targets in Bayesian inference. This includes, for instance, posterior means, standard deviations, and mean squared errors. Therefore, the corollary implies that even though $\hat{\pi }$ may not be the exact posterior, inference based on its samples remains valid to the second moments.

## Numerical results

### A Gaussian mixture distribution

We tested the ESGLD algorithm on a two-dimensional mixture Gaussian distribution:

10 $$\begin{aligned} \pi (\boldsymbol{x})=\frac{1}{2\pi \sigma } \sum _{i=1}^{20} w_i \exp \left\{ -\frac{1}{2\sigma ^2}(\boldsymbol{x}-\boldsymbol{\mu }_i)^T(\boldsymbol{x}-\boldsymbol{\mu }_i) \right\} , \end{aligned}$$

where σ=0.1 and $w_1=w_2=\cdots =w_{20}=0.05$, and the centers $\boldsymbol{\mu }_1, \boldsymbol{\mu }_2,\ldots , \boldsymbol{\mu }_{20}$ are randomly generated from the rectangle [0,10]×[0,10]; they are given in Table A1. This example creates a particularly challenging sampling landscape: each component has small variance, while several modes are widely separated, making transitions between them highly unlikely. Notably, these defining features — “small σ” and “well-separated modes” make $\pi (\boldsymbol{x})$ resemble a posterior distribution in a big-data regime, where a large sample size induces strong concentration and sharp multimodality.

Using this example, we compared ESGLD with both single-chain SGMCMC algorithms and population-based MCMC algorithms. The former include SGLD, SGHMC, SGNHT, preconditioned SGLD (pSGLD), momentum SGLD (MSGLD), and cyclical SGLD (cSGLD); the latter include replica-exchange SGLD (RESGLD), parallel tempering (PT), and evolutionary Monte Carlo (EMC). As suggested by the recent theoretical development in Zhou (2025), PT and EMC are expected to mix well on this problem with an appropriate choice of tuning parameters. Each algorithm was run from a random initialization in [0,1]×[0,1] for approximately the same CPU time. For the gradient-based methods, the learning rate was set to 0.001. See Supplement B.1 for full implementation details.

**Fig. 1:**
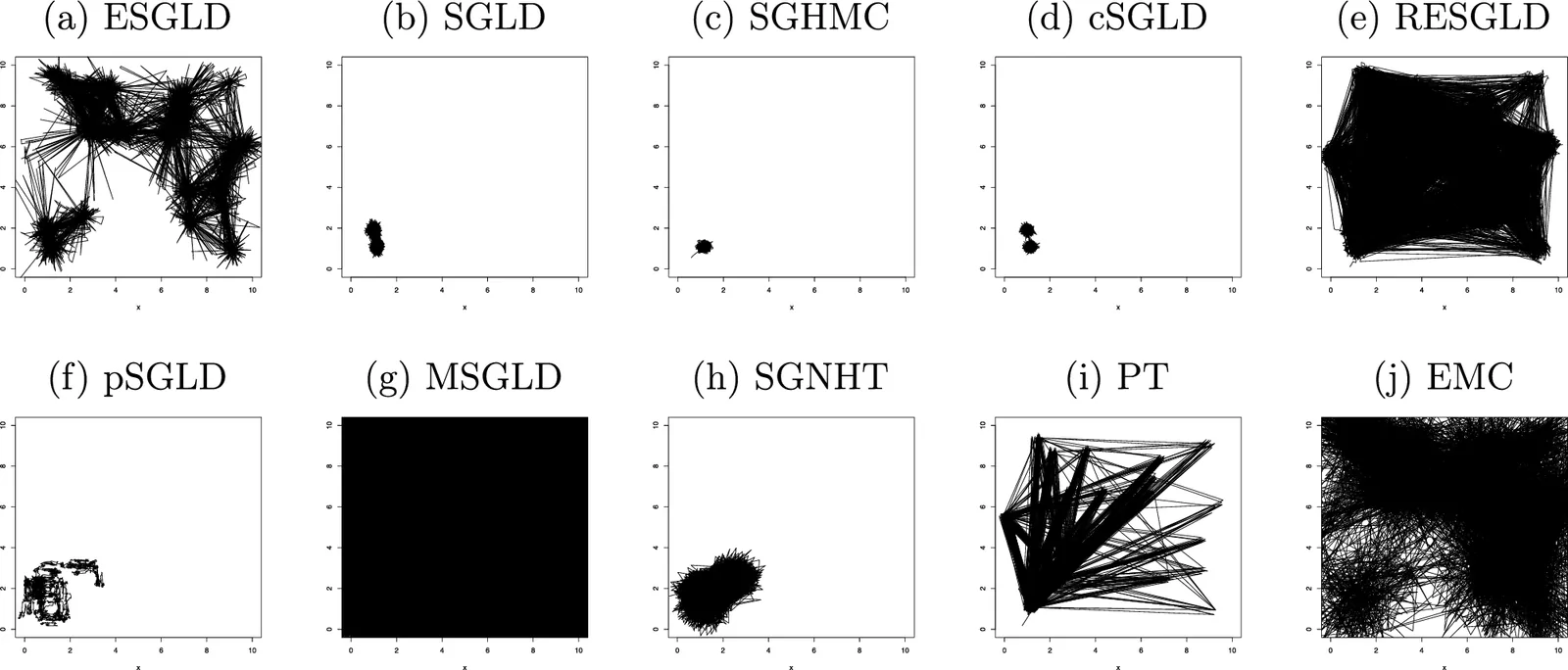
Sample paths generated by different algorithms, which are initialized uniformly within the square region [0,1]∗[0,1]

Figure 1 displays the sample paths produced by each algorithm over the same CPU time, corresponding to the first 8000 iterations of a single ESGLD run, or approximately 26.7% of a single run of each algorithm. As indicated by respective plots, SGLD, SGHMC, cSGLD, pSGLD, and SGNHT visited only a subset of modes.

The PT trajectory exhibits a hub-and-spike pattern induced by exchange moves. The lowest-temperature chain spends much of its time near the initial mode, while occasional accepted swaps transfer states discovered by higher-temperature chains to the lowest-temperature level. Because each swap simultaneously moves the previous cold-chain state to a higher-temperature chain, a subsequent exchange may return the cold chain to its original region, thereby producing repeated hub–spike–hub transitions.

The RESGLD trajectory indicates that it can visit all modes. However, our examination of the underlying samples shows that the upper-right and lower-right modes are visited less frequently than the others, a pattern that persists across repeated runs. Since these modes have relatively large $X_1$ values, their underrepresentation explains the downward bias in $\mathbb {E}(X_1)$. Their $X_2$ values lie at opposite ends of the state space, so their effects partially offset, leaving $\mathbb {E}(X_2)$ relatively close to the true value.

In contrast, ESGLD and EMC visit all modes within the same CPU budget and exhibit frequent long-distance transitions among well-separated modes. This demonstrates the effectiveness of the population-based snooker mechanism in overcoming local traps. In particular, ESGLD achieves such global exploration using stochastic-gradient updates without an acceptance-rejection step, making it more suitable for large-scale problems. MSGLD behaves differently from the other methods due to its adaptively biased drift: it is effective at exploring the global state space, but less effective at concentrating within individual modes.

Table 1 compares the estimated mean vector and covariance matrix produced by the different algorithms for the mixture distribution. Each estimate is computed by aggregating samples from 20 independent runs. Overall, ESGLD, PT, EMC, and RESGLD yield noticeably more accurate estimates of both the mean and covariance than the other methods. As noted above, however, ESGLD remains scalable to big-data settings while preserving strong mobility for cross-mode transitions.

**Table 1:** The mean vector and covariance matrix estimates produced by different algorithms for the mixture distribution (10): ‘SD’ denotes the standard deviation of the estimate, $(\mathbb {E}(X_1),\mathbb {E}(X_2))$ denotes the mean vector, and $\textrm{Var}(X_1)$, $\textrm{Var}(X2)$, and $\textrm{Cov}(X_1,X_2)$ denote three elements of the covariance matrix; the estimates closest to the true values are shown in bold

parameters	true	ESGLD	RESGLD	cSGLD	SGLD	SGHMC	pSGLD	MSGLD	SGNHT	PT	EMC
$\mathbb {E}(X_1)$	5.403	**5**.**477**	4.999	1.573	1.152	1.080	1.483	4.996	3.262	4.943	6.006
SD		0.619	0.298	0.031	0.328	0.060	0.910	0.006	2.022	0.832	0.252
$\mathbb {E}(X_2)$	5.493	5.637	5.307	1.850	1.552	1.491	2.614	**5**.**535**	3.434	5.207	6.063
SD		0.317	0.189	0.254	0.235	0.339	1.923	0.005	1.718	0.682	0.151
$\textrm{Var}(X_1)$	10.185	9.519	**10**.**324**	0.517	0.025	0.012	1.481	13.935	4.639	9.818	9.926
SD		0.809	0.253	0.015	0.001	0.002	0.552	0.016	3.921	0.690	0.612
$\textrm{Var}(X_2)$	7.535	7.124	8.872	0.386	0.181	0.070	2.079	10.564	3.922	**7**.**549**	8.286
SD		0.774	0.234	0.004	0.038	0.065	2.214	0.015	3.228	0.754	0.386
$\textrm{Cov}(X_1,X2)$	−0.327	−0.670	−0.806	0.324	−0.029	−0.011	−0.037	**−0.601**	−0.095	0.540	−2.271
SD		1.174	0.644	0.010	0.009	0.012	0.744	0.015	2.146	2.242	0.618

**Fig. 2:**
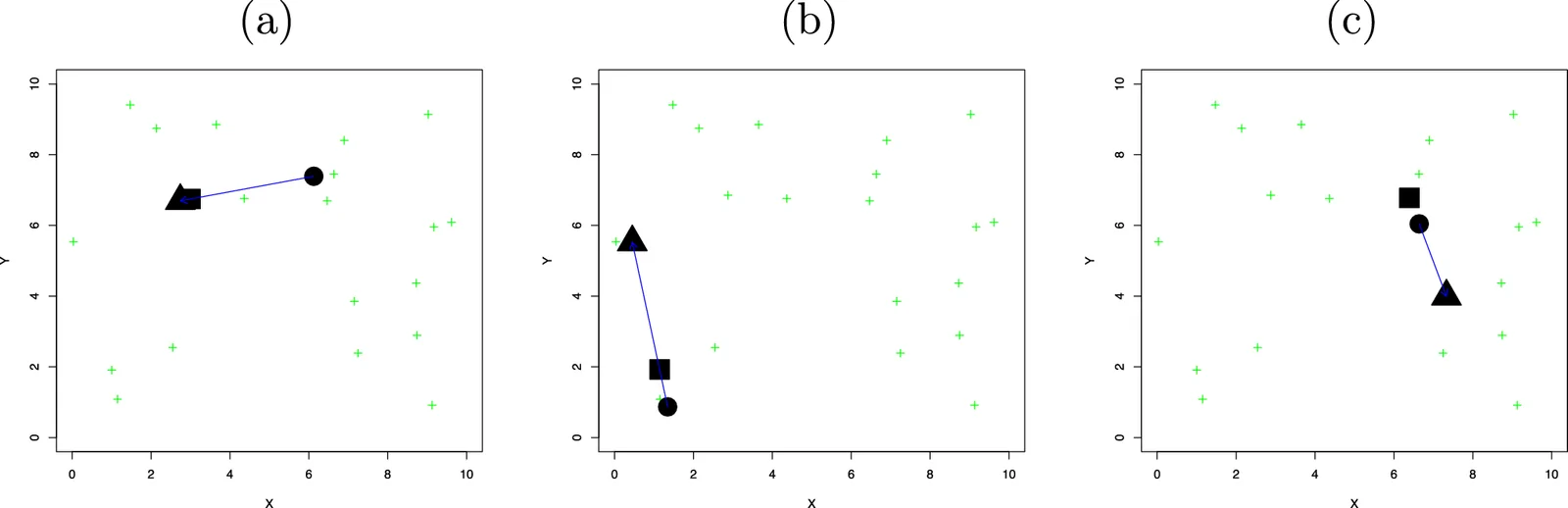
Mode transition types by the snooker operator, where the square denotes the anchor point, the round dot represents the current point, and the triangle indicates the new point: **a** transition to the mode where the anchor point is located, **b** transition to a mode in the direction towards the anchor point, and **c** transition to a mode in the direction away from the anchor point

Figure 2 illustrates three types of mode transitions achieved by the snooker operator in simulations of the example. In type (a), the transition is to the mode where the anchor chain is located; in type (b), transition is to a mode in the direction of the anchor point; and in type (c), the transition is to a mode in the direction away from the anchor point. As implied by the marginal density function (8), when two or more similar modes exist along the line $\boldsymbol{e}$, the snooker operator tends to transition into the mode farthest from the snooker point. Since multiple high-temperature chains are typically involved in ESGLD, it can be said that the algorithm makes transitions based on global information of the energy landscape, which accelerates its convergence toward the target distribution.

Additional numerical results for this example are provided in Supplement B.1, highlighting the sensitivity of ESGLD to the learning rate, population size (i.e., number of chains), temperature ladder, and mutation rate. Extensive empirical studies demonstrate that ESGLD is highly robust to variations in these hyperparameter settings. The reason for ESGLD’s stability across different experimental setups lies in the presence of the snooker operator, which enables frequent transitions across modes, effectively mitigating the impact of changes in tuning parameters.

### A distribution with long narrow energy ravines

Other than multimodal distributions, we tested ESGLD on a distribution with the energy landscape containing many long narrow energy ravines. This example is taken from Kim et al. (2021), with the data being generated from a nonlinear regression:

11 $$\begin{aligned}&y = f_{\boldsymbol{\theta }}(x)+\epsilon :=(x - 1)^2 + 2\sin (\theta _1 x) + \frac{1}{30}\theta _1 \nonumber \\&\quad \quad + \cos (\theta _2 x - 1) - \frac{1}{20}\theta _2+\epsilon , \end{aligned}$$

where $\epsilon \sim N(0,1)$ and *x* is uniformly distributed on the interval U[-2,4], and $\boldsymbol{\theta }=(\theta _1, \theta _2)^T=(20,10)^T$. We simulated five datasets from the regression independently, each consisting of N=10,000 observations. We consider *N* is large for this example, with the mini-batch strategy being used in simulating from the posterior distribution. See the Supplement B.2 for the detail.

**Fig. 3:**
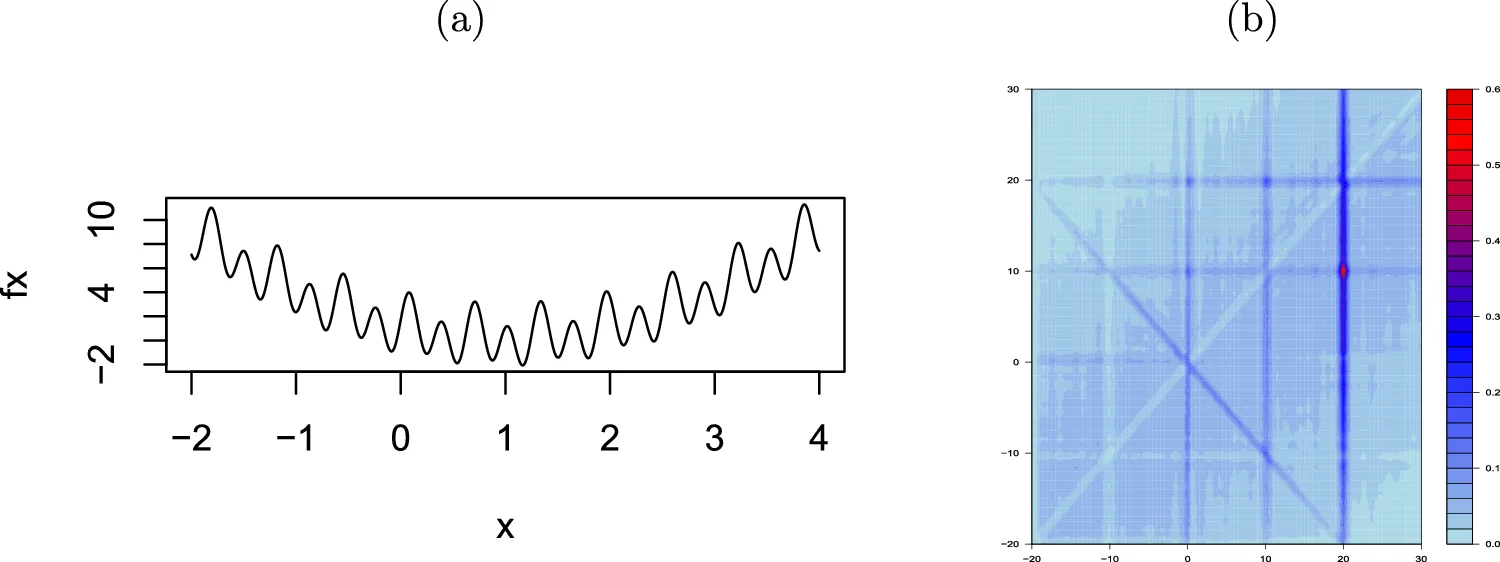
**a** The nonlinear regression curve $f_{\boldsymbol{\theta }}(x)$, with a global pattern determined by the quadratic term $(x-1)^2$ and the local bumps and pits determined by its other terms; **b** contour plot of the log-likelihood function

Figure 3 depicts the example, where plot (a) shows that the global pattern of the regression is determined by the quadratic term $(x-1)^2$, while the local bumps and pits are determined by the terms $2\sin (\theta _1 x) + \frac{1}{30}\theta _1 + \cos (\theta _2 x - 1) - \frac{1}{20}\theta _2$; and plot (b) shows the contour plot of the log-likelihood function (as a function of $\boldsymbol{\theta }$). The log-likelihood function has a global mode of (20,10), shown as the red spot, and many local modes taking shapes of long narrow ridges. These ridges become long narrow ravines in the energy landscape.

To conduct Bayesian analysis for the problem, we follow Kim et al. (2021) to set the prior distribution: $\theta _1 \sim N(0,1)$ and $\theta _2\sim N(0,1)$, which are *a priori* independent. This choice of prior distribution makes the problem even harder, discouraging the convergence toward the true parameter $\boldsymbol{\theta }=(20,10)$. Specifically, it biases $\boldsymbol{\theta }$ toward smaller values and encourages estimation of $f_{\boldsymbol{\theta }}(x)$ by the global pattern $(x-1)^2$. In simulations, we initialized the Markov chains by drawing $\theta _1$ and $\theta _2$ from *N*(0, 1) independently. The Markov chains need to trespass lots of misleading narrow ravines of different depth on the bumpy energy landscape before they reach the global optimum near (20,10). For this example, ESGLD has been compared with the SGLD, pSGLD, MSGLD, cSGLD, SGHMC, SGNHT, and RESGLD algorithms. All algorithms are stochastic-gradient–based and scalable to large datasets, and each was run for approximately the same CPU time. Refer to Supplement B.2 for the detailed experimental setup.

**Table 2:** Parameter estimates produced by different algorithms for regression (11): the smallest bias and SD values across methods are shown in bold

Dataset		ESGLD	RESGLD	SGHMC	pSGLD
		($\theta _1,\theta _2)$	$(\theta _1,\theta _2)$	$(\theta _1,\theta _2)$	$(\theta _1,\theta _2)$
Dataset 1	bias	**0.11, −0.3**	−5.43, −4.25	−11.86, −9.35	−4.85, −5.01
	SD	**3.24, 3.33**	22.02, 51.32	28.22, 19.32	30.48, 24.23
Dataset 2	bias	**−1.32, −0.25**	−8.72, −11.02	−11.19, −9.25	−7.84, −5.19
	SD	**4.22, 3.8**	18.7, 46.09	24.39, 19.03	30.84, 23.44
Dataset 3	bias	−0.76, 0.36	−8.22, −8.79	−11.39, −8.71	−4.92, −3.72
	SD	**1.87, 3.18**	23.6, 50.07	25.41, 19.9	32.81, 23.11
Dataset 4	bias	**−1.25, −0.13**	−8.52, −11.71	−11.95, −9.33	−7.96, −5.12
	SD	**3**.**47**, 4.82	31.14, 57.11	24.63, 18.48	31.37, 23.24
Dataset 5	bias	**−0.39, −1.71**	−6.75, −10.2	−12.66, −9.21	−8.96, −5.44
	SD	**1.13, 3.74**	20.79, 59.53	25.42, 18.69	28.41, 22.06

		MSGLD	SGLD	SGNHT	cSGLD
		($\theta _1,\theta _2)$	$(\theta _1,\theta _2)$	$(\theta _1,\theta _2)$	$(\theta _1,\theta _2)$
Dataset 1	bias	−1.86, −2.14	−4.42, −3.85	−13.43, −9.56	−9.77, −8.4
	SD	5.94, 4.5	72.37, 49.48	53.62, 36.27	33.29, 22.75
Dataset 2	bias	−7.05, −4.91	−3.46, −2.73	−2.14, −2.28	−8.02, −6.95
	SD	9.81, 6.81	66.08, 45.11	53.48, 36.35	33.88, 21.9
Dataset 3	bias	−3.15, −1.59	−1.38, −1.2	**0.56, 0.21**	−8.29, −6.94
	SD	8.58, 5.7	67.12, 46.06	47.59, 32.25	35.4, 21.82
Dataset 4	bias	−1.71, −1.56	−3.28, −2.46	−1.38, −1.66	−11.69, −9.1
	SD	4.02, **3**.**32**	68.69, 47.1	55.18, 37.43	36.14, 24.14
Dataset 5	bias	−3.06, −2.51	−3.93, −2.79	−5.98, −4.59	−5.81, −5.84
	SD	6.73, 6.16	62.5, 43.13	53.5, 36.19	37.65, 24.98

The comparison, see Table 2, indicates that ESGLD significantly outperforms other algorithms by achieving both the lowest bias and the lowest variance in parameter estimation. The slightly negative bias in the ESGLD estimate is attributed to our choice of the biased prior distribution $\boldsymbol{\theta }\sim N(0,I_2)$. Except for MSGLD, all other algorithms produced estimates with standard deviations larger than 10, indicating their sample paths were misled and consequently trapped in numerous energy ravines scattered across the sampling space. This is likely because these algorithms, except for RESGLD, rely solely on the local energy landscape for transitions. Although RESGLD can make transitions based on non-local information of the energy landscape, it can be inefficient in state swapping when the variation of the stochastic energy is high. As mentioned previously, ESGLD achieves mode transitions unfailingly through the snooker operator. Furthermore, the selection of anchor points is guided by global information of the energy landscape, which significantly accelerates the algorithm’s convergence. This is an exceptionally attractive feature for an MCMC algorithm.

### A mixture distribution with general shapes

Further, we evaluated the performance of ESGLD on a target with a more general geometry. Consider the 5-dimensional Gaussian mixture

$$ p N(\boldsymbol{\mu }_1, \Sigma _1)+(1-p) N(\boldsymbol{\mu }_2, \Sigma _2), $$

where p=2/3, $\boldsymbol{\mu }_1 = (0,0,0,0,0)^\top$ and $\boldsymbol{\mu }_2 = (1.5,1.5, 1.5,1.5,1.5)^\top$. We generated $\Sigma _1$ and $\Sigma _2$ via Cholesky factorization by sampling their lower-triangular factors with i.i.d. entries from $\textrm{Unif}(0,0.5)$, and then forming the corresponding covariance matrices:

$$\begin{aligned}&\Sigma _1 = \begin{bmatrix} 0.01762371 & 0.02470053 & 0.06028426 & 0.06270487 & 0.01367201 \\ 0.02470053 & 0.11665929 & 0.11337500 & 0.18251912 & 0.04444730 \\ 0.06028426 & 0.11337500 & 0.41815525 & 0.38910550 & 0.20997256 \\ 0.06270487 & 0.18251912 & 0.38910550 & 0.43216678 & 0.19179886 \\ 0.01367201 & 0.04444730 & 0.20997256 & 0.19179886 & 0.32144742 \end{bmatrix} \\&\Sigma _2 = \begin{bmatrix} 0.008545363 & 0.03246413 & 0.007767455 & 0.005969801 & 0.02554491 \\ 0.032464120 & 0.20550809 & 0.164790810 & 0.142139020 & 0.13128714 \\ 0.007767455 & 0.16479081 & 0.452304786 & 0.312477736 & 0.25897037 \\ 0.005969801 & 0.14213902 & 0.312477736 & 0.308210604 & 0.18146795 \\ 0.025544910 & 0.13128714 & 0.258970370 & 0.181467950 & 0.28446235 \end{bmatrix} \end{aligned}$$

The Bhattacharyya distance (Bhattacharyya 1943) between the two components is $D_B=54.932$, and the corresponding Bhattacharyya coefficient is $\exp (-D_B)=1.4\times 10^{-24}$, indicating essentially zero overlap.

For this example, we ran ESGLD with an ensemble size of 20, a temperature ladder equally spaced from 1 to 10, a learning rate of 0.001, an initial population drawn from $\textrm{Unif}([0,1]^5)$, and 30,000 iterations. We then clustered the samples at temperature level t=1 by fitting a two-component Gaussian mixture using the mclust R package (Fraley et al. 2024). We repeated the experiment 20 times, and summarize the resulting estimates of the mixture proportion *p* in Table 3.

For comparison, we also applied SGLD, SGHMC, SGNHT, pSGLD, cyclical SGLD, MSGLD, and RESGLD to the same example. All the algorithms are stochastic-gradient–based and scalable to large datasets. Each algorithm was run with the same CPU time as ESGLD, using learning rate 0.001, temperature fixed at 1, and the same initialization scheme. Afterward, each run was thinned to 30,000 samples for evaluation. Overall, ESGLD achieves performance comparable to RESGLD and MSGLD, and yields more accurate estimates than the remaining baselines.

**Table 3:** The mixture proportion estimates produced by different algorithms, where the estimate and its standard deviation (in parenthesis) are calculated by averaging over 20 independent runs, and MSE is calculated as $(\hat{p}-0.667)^2+SD^2$

	ESGLD	RESGLD	SGHMC	pSGLD	MSGLD	SGLD	SGNHT	cSGLD
$\hat{p}$	0.647	0.654	0.767	0.523	0.667	0.583	0.597	0.608
SD	(0.015)	(0.022)	(0.049)	(0.011)	(0.027)	(0.016)	(0.021)	(0.023)
MSE($\times 10^{-4}$)	6.25	6.53	124.1	208.57	7.29	73.12	53.41	40.1

### Towards better generalization of neural networks

Deep learning has recently achieved remarkable success across various applications, promoting extensive research into generalization analysis for deep neural networks. Researchers have employed diverse methods for this analysis, including VC-dimension (Bartlett et al. 2019), Rademacher complexity (Arora et al. 2019), and energy landscapes (Wu et al. 2017; Keskar and Socher 2017; He et al. 2019). Empirical observations suggest that wide energy valleys are often associated with better generalization performance. Building upon this insight, some training methods have been proposed to enhance the generalization of deep neural networks. For example, Chaudhari et al. (2017) introduced the entropy-SGD method, which biases gradient descent towards wide valleys.

From a Bayesian perspective, neural network training is to sample from a scaled posterior distribution:

12 $$\begin{aligned} \pi (\boldsymbol{\theta }|\boldsymbol{x},\boldsymbol{y}) \propto \exp \{-U(\boldsymbol{y},\boldsymbol{x};\boldsymbol{\theta })/\tau \}, \end{aligned}$$

where $U(\boldsymbol{y},\boldsymbol{x};\boldsymbol{\theta })=-\log f(\boldsymbol{y}|\boldsymbol{x},\boldsymbol{\theta }) -\log \pi (\boldsymbol{\theta })$ denotes the negative log-posterior distribution, $\pi (\boldsymbol{\theta })$ denotes the prior distribution of $\boldsymbol{\theta }$, and τ denotes the target temperature. In training neural networks using ESGLD, we typically set the target temperature to a value smaller than 1 to facilitate the identification of local minimum energy models, except when the training sample size is large. It is worth noting that setting τ to a small value is approximately equivalent to increase the sample size of the dataset.

Suppose that the posterior distribution contains multiple modes. The choice of τ determines the relative mass of each mode. That is, different values of τ assign different masses to the modes, allowing a MCMC sampler to focus on different modes as τ varies. For better generalization of the neural network, the choice of τ may vary depending on the problem, biasing the sampler towards the modes that yield better predictions. However, selecting τ is not the focus of this paper. Instead, for a given value of τ, we are interested in whether the models (corresponding to different modes of (12) sampled by ESGLD can provide better Bayesian model averaging (BMA) predictions compared to those generated by other samplers. Our interest stems from the predictive property of BMA, which, as shown in Le and Clarke (2022), can asymptotically perform as well or better than any of its components in squared error loss. Based on this theory, we conjecture that a MCMC sampler with better mixing can typically yield improved BMA predictions.

We tested this conjecture using 10 UCI datasets (available at https://archive.ics.uci.edu/datasets), see Table 4, each modeled by a small neural network. Each neural network has a single hidden layer, with 50 hidden units if the training sample size is smaller than 10,000 and 100 hidden units otherwise. The prior for all neural network was set as $\pi (\boldsymbol{\theta }) \propto 1$. ESGLD operates on a ladder of temperatures. As expected, it yielded different predictions at different temperature levels. For the datasets ‘Real Estate’ and ‘Protein’, we reported the prediction results at τ=0.08 and 1.14, respectively; For the remaining datasets, we reported the prediction results at τ=0.01. The detailed experimental setup can be found in Supplement B.3. The numerical results are summarized in Table 4. For comparison, we also applied SGLD and RESGLD to train the neural networks at respective temperature levels for each dataset. As expected, ESGLD consistently produced lower prediction errors (in squared error loss) compared to RESGLD and SGLD across these datasets. We also list in the table the CPU time it costs with an eight-core CPU with four 4.05 GHz and four 2.75 GHz cores

**Table 4:** Comparison of mean squared prediction errors produced by ESGLD, RESGLD, and SGLD for neural network models: $^\dag$ scaled by a factor of $10^4$, $^\ddag$ scaled by a factor of $10^5$, $^*$ scaled by a factor of $10^2$, *p* and *d* represent the input dimension and parameter dimension of the neural network, respectively, and the smallest mean squared prediction errors across methods are shown in bold

dataset	*p*	*N*	*d*	runtime (sec)	ESGLD	RESGLD	SGLD
Boston	13	506	751	2	$\mathbf{1.363}\pm 0.093$	1.390±0.111	1.502±0.153
Energy	8	768	501	3	$\mathbf{1.587} \pm 0.145$	1.688±0.383	1.839±0.023
kin8nm$^\dag$	8	8,192	501	31	$\mathbf{4.384}\pm 2.906$	13.246±4.109	12.373±3.714
Concrete	8	1,030	501	3	$\mathbf{5.288} \pm 0.346$	5.312±0.522	5.439±0.425
Abalone	8	4,180	501	14	$\mathbf{0.142} \pm 0.009$	0.147±0.0028	0.175±0.007
Real estate	6	415	401	2	$\mathbf{1.883} \pm 0.445$	2.052±0.091	2.106±0.139
Electric$^\ddag$	12	10,000	1401	38	$\mathbf{8.137} \pm 1.073$	8.423±5.06	11.079±5.053
Qsar$^*$	6	908	401	3	$\mathbf{2.240} \pm 0.160$	2.760±0.160	2.910±0.170
Student	4	649	301	36	$\mathbf{1.685} \pm 0.166$	1.789±0.156	1.936±0.122
Protein$^*$	9	45,730	1101	312	$\mathbf{7.547} \pm 0.010$	7.550±0.014	7.551±0.015

Later, we endeavored to further enhance the performance of ESGLD by implementing the mutation step using SGHMC, resulting in the ESGHMC algorithm. Since SGHMC incorporates momentum terms, it generally achieves better mixing than SGLD. Therefore, we anticipate that ESGHMC will also achieve better mixing than ESGLD. Table 5 compares the performance of ESGHMC, replica exchange SGHMC (RESGHMC), and SGHMC algorithms on the UCI datasets. Indeed, for some datasets, such as ’Electric’ and ’Student’, ESGHMC significantly outperforms ESGLD. Notably, ESGLD consistently outperforms SGLD, and ESGHMC consistently outperforms SGHMC across all the datasets, highlighting the positive impact of the snooker operator on the mixing of stochastic gradient MCMC.

**Table 5:** Comparison of mean squared prediction errors produced by ESGHMC, RESGHMC, and SGHMC for neural network models: the smallest mean squared prediction errors across methods are shown in bold, $^\dag$ scaled by a factor of $10^4$, $^\ddag$ scaled by a factor of $10^5$, $^*$ scaled by a factor of $10^2$

dataset	ESGHMC	RESGHMC	SGHMC
Boston	$\mathbf{1.418}\pm 0.081$	1.559±0.226	1.612±0.163
Energy	$\mathbf{1.444} \pm 0.387$	1.632±0.412	1.826±0.020
kin8nm$^\dag$	$\mathbf{3.460}\pm 2.532$	13.574±3.654	11.573±2.680
Concrete	$\mathbf{5.500} \pm 0.147$	5.678±0.106	5.694±0.074
Abalone	$\mathbf{0.122} \pm 0.012$	0.140±0.002	0.139±0.003
Real estate	$\mathbf{2.058} \pm 0.160$	2.144±0.186	2.108±0.181
Electric$^\ddag$	$\mathbf{2.326} \pm 1.073$	3.408±0.918	2.415±0.645
Qsar$^*$	$\mathbf{1.960} \pm 0.170$	2.200±0.090	2.170±0.680
Student	$\mathbf{1.198} \pm 0.348$	1.269±0.523	1.295±1.003
Protein$^*$	$\mathbf{7.549} \pm 0.007$	7.551±0.015	7.553±0.013

We have also tested the ESGHMC algorithm in simulations of extremely high-dimensional distributions, training ResNet 20, ResNet32, and ResNet56 (He et al. 2016) for the CIFAR100 data (available at https://www.cs.toronto.edu/~kriz/cifar.htm). The dimensions of the three models are 0.27 millions, 0.46 millions, and 0.85 millions, respectively. For these highly over-parameterized models, nearly all local energy minima are global energy minima, so local trapping is no longer a concern for model training and different MCMC algorithms may perform similarly. However, our experiments show that even for those high-dimensional distributions where the parameter dimension *d* is much higher than the training sample size *N*, ESGHMC still enhances the performance of SGHMC. We omitted detailed numerical results for these models.

## Discussion

In this paper, we have successfully addressed the local trap issue encountered by single-chain stochastic gradient MCMC algorithms, such as SGLD and SGHMC (Mangoubi et al. 2018), in big data scenarios. The proposed algorithm works with a population of Markov chains, each at a different temperature, and different chains interact through the snooker operator. Incorporating chains at varying temperatures strikes a balance between exploration and exploitation in the simulation, while the snooker operator enables unfailingly, non-local mode transitions, thereby effectively escaping local traps. By leveraging information from the entire energy landscape, which is gathered from the population of Markov chains, in selecting anchor points, the snooker operator accelerates convergence of the simulation, particularly when the energy landscape is rugged. Our numerical results show that the proposed algorithm is effective not only for low-dimensional problems but also for high-dimensional challenges, such as neural networks.

The proposed algorithm has a highly flexible framework. In Algorithm 1, we select anchor points at each iteration from current samples of other chains. However, this is unnecessary. As implied by Lemma 4.1, the algorithm remains valid as long as the anchor point is selected independently of the current chain. Given the mutual independence among different chains in the population, one may select the anchor point from all historical samples, rather than only the current samples, generated by other chains. This strategy will allow the snooker moves to leverage more information about the energy landscape and enable the proposed algorithm to work with a smaller population, thereby further enhancing its efficiency. Apart from SGLD, the mutations can be performed using other single-chain SGMCMC algorithms, such as SGHMC and SGNHT. As discussed in Nemeth and Fearnhead (2019), SGHMC and SGNHT can achieve faster convergence than SGLD, especially when the gradient noise is minimal.

Additionally, different chains can utilize different algorithms for performing mutations.

The theory developed in this paper has many implications for further developments of SGMCMC algorithms. Specifically, the snooker operator can be regarded as a one-dimensional update conditioned on a given direction $\boldsymbol{e}$, akin to the conditional update employed by the Gibbs sampler (Geman and Geman 1984). That is, our theory lays the groundwork for integrating low- and high-dimensional moves in SGMCMC simulations, leading to enhanced convergence especially for multimodal distributions. In particular, the proposed ESGLD algorithm is well-suited for inference with large-scale datasets. Although it does not guarantee convergence for a fixed number of training samples, as is common among stochastic gradient MCMC algorithms, it offers the advantage of traversing freely across rugged energy landscapes. As indicated by our numerical studies, this global exploration capability can yield more accurate posterior estimates compared to algorithms that achieve only faster local convergence.

Finally, we note that this paper achieves significantly better convergence results compared to Raginsky et al. (2017). Specifically, in the latter, if the gradient noise term δ>0 and kη is reasonably large, the 2-Wasserstein distance cannot converge to zero even as $N\rightarrow \infty$. However, under slightly more restrictive conditions – namely, those imposed on *M*, β, $C_g$, and *B* – which are generally satisfied in the big data regime, we establish the convergence of the 2-Wasserstein distance toward zero. This, combined with the algorithm’s immunity to local traps, makes it a promising tool for Bayesian inference of big data.

## Supplementary Information

Below is the link to the electronic supplementary material.

Supplementary file 1 (pdf 2458 KB)

## Data Availability

No datasets were generated or analysed during the current study.
