# Supplementary material for: Evolutionary stochastic gradient Langevin dynamics: overcoming local traps in large-scale Bayesian sampling
Source: Stat Comput. 2026 Nov 5;36(6):258. doi: 10.1007/s11222-026-11020-6 (PMC13740107; doi:10.1007/s11222-026-11020-6)
Supplement: Supplementary file 1 [file 11222_2026_11020_MOESM1_ESM.pdf]

Supplementary Material for "Evolutionary Stochastic Gradient Langevin Dynamics: Overcoming Local Traps in Large-Scale Bayesian Sampling"

Yushu Huang† and Faming Liang*

August 18, 2026

A Theoretical Proofs

A.1 Proof of Proposition 1

Proof. Recall that the target joint distribution of the population is given by

$$\pi_{\boldsymbol{\tau}}(\boldsymbol{\theta}^{(1)}, \ldots, \boldsymbol{\theta}^{(m)}) \;=\; \prod_{i=1}^{m} \pi_{\tau_i}(\boldsymbol{\theta}^{(i)}),$$

where $\pi_{\tau_i}(\boldsymbol{\theta}) \propto \exp\{-U(\boldsymbol{\theta})/\tau_i\}$.

We prove that $\pi_{\boldsymbol{\tau}}$ is invariant for the population update described in Proposition 1. The key point is that the snooker anchor point may be *different at different update times*: Lemma 4.1 requires only that, within a single snooker move, the anchor point is treated as fixed, and (for random anchors) independent of the current state of the chain being updated.

Step 1: Each Langevin diffusion marginally preserves π_{τ_i}. Under condition (7) and the regularity conditions in Roberts and Tweedie (1996), for each i the Langevin diffusion

$$d\boldsymbol{\theta}_t^{(i)} \;=\; -\nabla_{\boldsymbol{\theta}} U(\boldsymbol{\theta}_t^{(i)})\, dt + \sqrt{2\tau_i}\, dW_t^{(i)}$$

admits π_{τ_i} as an invariant distribution.

*Correspondence author: Faming Liang, Department of Statistics, Purdue University, West Lafayette, IN 47907, USA; email: fmliang@purdue.edu. † Department of Statistics, Purdue University, West Lafayette, IN 47907, USA; email: huan1455@purdue.edu.

Step 2: A snooker move preserves π_{τ_i} even with a time-varying anchor. Fix an index i. Consider one snooker operation applied to $\boldsymbol{\theta}^{(i)}$. Let $\boldsymbol{\phi} \in \mathbb{R}^d$ be the anchor point used in *this* snooker move. Note that the anchor point $\boldsymbol{\phi}$ is only required to be fixed within a given snooker move; it may vary across iterations. Define $\boldsymbol{e} = (\boldsymbol{\theta}^{(i)} - \boldsymbol{\phi})/\|\boldsymbol{\theta}^{(i)} - \boldsymbol{\phi}\|$ and draw r from

$$f(r) \propto |r|^{d-1} \pi_{\tau_i}(\phi + r\boldsymbol{e}),$$

then set $\boldsymbol{\vartheta}^{(i)} = \boldsymbol{\phi} + r\boldsymbol{e}$. By Lemma 4.1,

$$\boldsymbol{\theta}^{(i)} \sim \pi_{\tau_i} \implies \boldsymbol{\vartheta}^{(i)} \sim \pi_{\tau_i}.$$

Moreover, Lemma 4.1 further states that if $\boldsymbol{\phi}$ is generated independently of $\boldsymbol{\theta}^{(i)}$, then $\boldsymbol{\vartheta}^{(i)}$ is independent of $\boldsymbol{\phi}$ and has density π_{τ_i}.

Thus, for invariance it is not necessary that the anchor be the same across iterations; it may vary from snooker move to snooker move, as long as it is independent of the current chain at the moment that chain is updated.

Step 3: Invariance of the joint *law π_τ for the population update.* Consider one discrete update time (one of the times $\{t\eta^{(i)}\}$). Let $\Theta = (\boldsymbol{\theta}^{(1)}, \dots, \boldsymbol{\theta}^{(m)})$ denote the pre-update population state. At this time, a random subset $\mathcal{I} \subset \{1, \dots, m\}$ of size pm is selected to undergo Langevin diffusion; the remaining indices $\mathcal{I}^c$ undergo snooker moves. (As stated in Proposition 1, these updates are performed independently across chains.)

Assume $\Theta \sim \pi_\tau$. Then the coordinates are independent under π_τ. For each $i \in \mathcal{I}^c$, let the anchor Φ_i be selected using only the other coordinates (e.g., by sampling one of the other chains, or from a subpopulation as in Algorithm 1), and treat Φ_i as fixed during the snooker move for chain i. Because Φ_i is measurable with respect to $\Theta_{-i} := \{\boldsymbol{\theta}^{(j)} : j \neq i\}$, we have $\Phi_i \perp\!\!\!\perp \boldsymbol{\theta}^{(i)}$ under π_τ. Therefore, by Step 2, after the snooker update the new state of chain i has marginal π_{τ_i}, and is independent of Φ_i. For each $i \in \mathcal{I}$, Step 1 shows that the Langevin diffusion update preserves π_{τ_i}.

Since (i) each chain is updated independently given the pre-update state and the selection mechanism, and (ii) each updated coordinate has the correct marginal and is independent of the random anchor used to generate it, the post-update population again has joint law $\prod_{i=1}^m \pi_{\tau_i} = \pi_\tau$.

Step 4: Conclusion (and the warm-up assumption). The above shows that π_τ is invariant for the population update kernel at the discrete update times. During the warm-up period, the chains evolve by Langevin diffusion, which converges to the corresponding π_{τ_i} (Roberts and Tweedie, 1996). After warm-up, applying the population update repeatedly preserves π_τ. Hence $\pi_\tau(\boldsymbol{\theta}^{(1)}, \dots, \boldsymbol{\theta}^{(m)})$ is invariant with respect to the snooker–Langevin diffusion described in Proposition 1. □

Remark A.1. *When* $(1-p)m$ *chains are updated simultaneously, the algorithm remains valid provided that the anchor samples are chosen appropriately so that they are independent of the chains being updated. Let* I^c *denote the set of chains being updated by the snooker operator, and suppose that the anchor points used for these updates are drawn only from the chains outside* I^c*. Since the population follows the product distribution, the current samples* $\{\theta_0^{(i)} : i \in I^c\}$ *are mutually independent and are also independent of the anchor samples.*

Conditioning on the selected anchor points, Lemma 4.1 implies that each snooker update produces $\theta_1^{(i)} \sim \pi_{\tau_i}$*, and, importantly, this conditional distribution does not depend on the anchor sample. Moreover, since the snooker updates use independent random variables across chains, the updated samples* $\{\theta_1^{(i)} : i \in I^c\}$ *are conditionally independent. Therefore, for any measurable sets* $\{A_i : i \in I^c\}$*,*

$$\Pr\left(\theta_1^{(i)} \in A_i,\ i \in I^c \,\middle|\, \textit{anchor samples}\right) = \prod_{i \in I^c} \pi_{\tau_i}(A_i).$$

Integrating with respect to the marginal distribution of anchor samples yield

$$\Pr\left(\theta_1^{(i)} \in A_i,\ i \in I^c\right) = \prod_{i \in I^c} \pi_{\tau_i}(A_i).$$

Hence, the simultaneously updated chains remain mutually independent, with each retaining its desired marginal distribution.

A.2 Two Useful Lemmas

Before considering the moments of $\pi(\boldsymbol{\theta}|\boldsymbol{x}_1, \ldots, \boldsymbol{x}_N)$, let's introduce a widely known theoretical result: integrability implies finite almost surely.

Lemma A.2. *Suppose that* f *is integrable with respect to a probability measure* μ *on the space* $\mathcal{X}$*, i.e.,* $\int_{\mathcal{X}} |f(\boldsymbol{x})|\mu(d\boldsymbol{x}) < C$ *for some constant* $C > 0$*. Then* $f(\boldsymbol{x})$ *is finite on* $\mathcal{X}$ *almost surely (a.s.).*

Proof. Define $S_n = \{\boldsymbol{x} : |f(\boldsymbol{x})| > n\} \cap \mathcal{X}$ for $n = 0, 1, \ldots$. Then $S = \cap_n S_n$ is the set where f goes to infinity. By the construction, we have

$$n\mu(S_n) \leq \int_{S_n} |f(\boldsymbol{x})|\mu(d\boldsymbol{x}) \leq \int_{\mathcal{X}} |f(\boldsymbol{x})|\mu(d\boldsymbol{x}) < C.$$

Since $S \subset S_n$, $0 \leq \mu(S) \leq \mu(S_n) \leq C/n$, which holds for all n. Let $n \to \infty$, we have $\mu(S) \to 0$; that is, f is finite on $\mathcal{X}$ almost surely. □

The proof of Theorem 4.4 involves the moment $\mathbb{E}(|r|^4)$. The existence of $\mathbb{E}(|r|^4)$ is implied by Assumption 1, with the justification given as follows. Let's define $V(s) := U(s\boldsymbol{\theta})$. Then $\frac{dV(s)}{ds}\Big|_{s=1} =$

$\boldsymbol{\theta}^T \nabla U(s\boldsymbol{\theta})|_{s=1} = \boldsymbol{\theta}^T \nabla U(\boldsymbol{\theta})$ and $\int_0^1 dV(s) = U(\boldsymbol{\theta}) - U(0)$, where $U(0)$ is assumed to be finite. Similarly, the right side of the dissipativity inequality can also be considered as a function of $s\boldsymbol{\theta}$ attained at $s = 1$. With the line integration method, it is easy to derive that

$$U(\boldsymbol{\theta}) \geq M^{\dagger} \|\boldsymbol{\theta}\|^2 + \beta^{\dagger},$$

for some constants $M^{\dagger} > 0$ and $\beta^{\dagger} \in \mathbb{R}$. Therefore, $\pi(\boldsymbol{\theta}|\boldsymbol{x}_1, \ldots, \boldsymbol{x}_N)$ is zero-concentrated with the tail decaying like Gaussian or faster, and it has finite moments of any order.

Let $\boldsymbol{e}$ be a unit vector, and let $\boldsymbol{\theta}$ be expressed as $\boldsymbol{\theta} = \boldsymbol{\phi} + r\boldsymbol{e}$. Without loss of generality, we let $\boldsymbol{\phi} = \mathbf{0}$. By the boundedness of the moments of $\pi(\boldsymbol{\theta}|\boldsymbol{x}_1, \ldots, \boldsymbol{x}_N)$, there exists a constant $M_r > 0$ such that

$$\mathbb{E}(\|\boldsymbol{\theta}\|^4) = \mathbb{E}\left[\mathbb{E}_{f(r)}(\|\boldsymbol{\theta}\|^4|\boldsymbol{e})\right] = \mathbb{E}\left[\mathbb{E}_{f(r)}(\|r\boldsymbol{e}\|^4|\boldsymbol{e})\right] = \int \left(\int |r|^4 f(r) dr\right) \mu(d\boldsymbol{e}) < M_r, \tag{A.1}$$

where $f(r)$, as given in Lemma 4.1, denotes the conditional distribution of $\boldsymbol{\theta}$ given $\boldsymbol{\phi}$ and $\boldsymbol{e}$, and $\mu(\boldsymbol{e})$ denotes a probability measure of $\boldsymbol{e}$. By Lemma A.2, $\|\boldsymbol{\theta}\|$ is almost surely finite with respect to $\pi(\boldsymbol{\theta}|\boldsymbol{x}_1, \ldots, \boldsymbol{x}_N)$; $\int |r|^4 f(r) dr$ is almost surely finite with respect to μ; and $|r|$ is almost surely finite with respect to ν, a joint probability measure of $\boldsymbol{e}$ and r. In other words, for any $\varepsilon > 0$, there exist constants $M_{\varepsilon} > 0$ and $B_{\varepsilon} > 0$ such that

$$P\left(\int |r|^4 f(r) dr \leq M_{\varepsilon}\right) \geq 1 - \varepsilon, \tag{A.2}$$

$$P(|r| < B_{\varepsilon}) \geq 1 - \varepsilon. \tag{A.3}$$

The next lemma concerns the uniform L^2-bounds established in Raginsky et al. (2017) for the SGLD sampler and Langevin diffusion.

Lemma A.3. *(Lemma 3; Raginsky et al. (2017)) Suppose Assumptions 1-5 hold, and choose* $\eta \in (0, \frac{M}{2C_g^2})$ *if* $M < 2C_g$, *and* $\eta \in (0, \frac{M-\sqrt{M^2-4C_g^2}}{4C_g^2}) \cup (\frac{M-\sqrt{M^2-4C_g^2}}{4C_g^2}, \frac{M}{2C_g^2})$ *if* $M \geq 2C_g$. *Then, for the SGLD sampler,*

$$\sup_{k \geq 0} \mathbb{E}\|\hat{\boldsymbol{\theta}}_k\|^2 \leq \kappa_0 + \frac{2}{M}(\beta + 2B^2 + d\tau), \tag{A.4}$$

and, for Langevin diffusion,

$$\mathbb{E}\|\boldsymbol{\theta}_t\|^2 \leq \kappa_0 + \frac{\beta + d\tau}{M}, \tag{A.5}$$

where κ_0 *denotes a constant related to the initial value* $\hat{\boldsymbol{\theta}}_0$.

Suppose that the first k iterations of the ESGLD sampler are run with SGLD for the warming-up process. By (A.4), $\mathbb{E}\|\hat{\boldsymbol{\theta}}_k\|^2$ is upper bounded, i.e., many samples lie in a region around zero.

Furthermore, by Assumptions 1, $\pi(\boldsymbol{\theta}|\boldsymbol{x}_1,\ldots,\boldsymbol{x}_N)$ is zero-concentrated with the tail decaying like Gaussian or faster. Therefore, with dominating probability, $\boldsymbol{\phi}$ can be contained in a compact set. That is, for any $\varepsilon > 0$, there exists a constant $m_{\boldsymbol{\phi}} > 0$ such that

$$P(\|\boldsymbol{\phi}\| \leq m_{\boldsymbol{\phi}}) \geq 1 - \varepsilon. \tag{A.6}$$

Recall that we select the anchor point from a subpopulation of $\{\hat{\boldsymbol{\theta}}_k^{(i)} : i = 1, 2, \ldots, m\}$ according to a tempering distribution as specified in Algorithm 1.

Under the assumption that (A.6) holds for iteration k, we will be able to show that the SGLD sampler performed in the snooker moves also satisfies Assumptions 1-5, see Lemma 4.2 and Lemma 4.3 for the smoothness and dissipativity, respectively. Consequently, we will be able to show that $\mathbb{E}\|\hat{\boldsymbol{\theta}}_{k+1}\|^2$ is upper bounded, and thus (A.6) holds for iteration $k+1$ as well. Therefore, by induction, (A.6) holds uniformly over all iterations of the ESGLD sampler. Note that the choice of $\boldsymbol{\phi}$ is essentially controlled by the dissipativity condition.

A.3 Proof of Lemma 4.2

Proof. For any point $\hat{\boldsymbol{\theta}}_k$, any anchor point $\boldsymbol{\phi}$, and any $r \in (-\infty, -a] \cup [a, \infty)$, we have

$$\begin{aligned} \nabla_r \hat{U}_r(r) &= -\nabla_r \log\left(|r|^{d-1}\pi\left(\boldsymbol{\phi} + r\hat{\boldsymbol{e}}\right)\right) \\ &= \frac{-d+1}{|r|} + \hat{\boldsymbol{e}}^T \nabla_{\boldsymbol{\theta}} U\left(\boldsymbol{\phi} + r\hat{\boldsymbol{e}}\right), \end{aligned} \tag{A.7}$$

where $\hat{\boldsymbol{e}} = \frac{\hat{\boldsymbol{\theta}}_k - \boldsymbol{\phi}}{\|\hat{\boldsymbol{\theta}}_k - \boldsymbol{\phi}\|}$. Therefore, for any $r_1, r_2 \in (-\infty, -a] \cup [a, +\infty)$,

$$\begin{aligned} &|\nabla_r \hat{U}_r(r_1) - \nabla_r \hat{U}_r(r_2)| \\ &= \left| \frac{-d+1}{|r_1|} + \hat{\boldsymbol{e}}^T \nabla_{\boldsymbol{\theta}} U\left(\boldsymbol{\phi} + r_1\hat{\boldsymbol{e}}\right) - \frac{-d+1}{|r_2|} - \hat{\boldsymbol{e}}^T \nabla_{\boldsymbol{\theta}} U\left(\boldsymbol{\phi} + r_2\hat{\boldsymbol{e}}\right) \right| \\ &\leq \left| (d-1)\frac{r_1 - r_2}{r_1 r_2} \right| + \|\hat{\boldsymbol{e}}\| \, \|\nabla_{\boldsymbol{\theta}} U(\boldsymbol{\phi} + r_1\hat{\boldsymbol{e}}) - \nabla_{\boldsymbol{\theta}} U(\boldsymbol{\phi} + r_2\hat{\boldsymbol{e}})\| \\ &\leq \frac{d-1}{a^2}|r_1 - r_2| + C_g \,\|(r_1 - r_2)\hat{\boldsymbol{e}}\| \\ &\leq (C_g + \frac{d-1}{a^2})|r_1 - r_2|, \end{aligned}$$

i.e., $\hat{U}_r$ is $C_{\boldsymbol{\phi}}$-smooth on $(-\infty, a] \cup [a, \infty)$ by defining

$$C_{\boldsymbol{\phi}} := C_g + \frac{d-1}{a^2} \asymp C_g. \tag{A.8}$$

In simulations, this can be realized by drawing r outside the interval $(-a, a)$. Since $f(0) = 0$, restricting the sample space of r to $(-\infty, -a] \cup [a, \infty)$ for a tiny value of a will not affect much the property of the snooker operator.

We can interpret the above result in a probabilistic way. As discussed in the Supplement A.2, under Assumption 1, all the anchor points used in the ESGLD sampler can be contained in a compact space (denoted by $\mathcal{S}_{\boldsymbol{\phi}}$) with dominating probability, see (A.6). Conditioned on this, for any $\varepsilon' > 0$, there exists a common value of a such that $\hat{U}_r$ is $C_{\boldsymbol{\phi}}$-smooth on $(-\infty, a] \cup [a, \infty)$, with probability $\int_{-\infty}^{a} \hat{f}(r)dr + \int_{a}^{\infty} \hat{f}(r)dr \geq 1 - \varepsilon'$, for any $\boldsymbol{\phi}$ and $\hat{\boldsymbol{e}}$. Note that each direction $\hat{\boldsymbol{e}}$ can be determined by $\boldsymbol{\phi}$ and another point in the same compact space $\mathcal{S}_{\boldsymbol{\phi}}$. Therefore, with dominating probability (no smaller than $(1-\varepsilon)(1-\varepsilon')$, which is $(1-\varepsilon)^2$ if we set $\varepsilon' = \varepsilon$), $\hat{U}_r$ is $C_{\boldsymbol{\phi}}$-smooth. In particular, the result holds uniformly with respect to the selected anchor point $\boldsymbol{\phi}$ and sampling direction $\hat{\boldsymbol{e}}$. □

A.4 Proof of Lemma 4.3

Proof. Let $\hat{\boldsymbol{e}} = \frac{\hat{\boldsymbol{\theta}}_k - \boldsymbol{\phi}}{\|\hat{\boldsymbol{\theta}}_k - \boldsymbol{\phi}\|}$. By (A.7), we have $\langle r, \nabla_r \hat{U}_r \rangle = (-d+1)r/|r| + r\hat{\boldsymbol{e}}^T \nabla_{\boldsymbol{\theta}} U(\boldsymbol{\phi} + r\hat{\boldsymbol{e}})$ as r is a scalar. Since $d \geq 1$, the following holds on $(-\infty, -a] \cup [a, \infty)$:

$$
\begin{aligned}
r\nabla_r \hat{U}(r) &\geq (-d+1) + r\hat{\boldsymbol{e}}^T (\nabla_{\boldsymbol{\theta}} U(\boldsymbol{\phi} + r\hat{\boldsymbol{e}}) \\
&= (-d+1) + r\hat{\boldsymbol{e}}^T \Big\{ \nabla_{\boldsymbol{\theta}} U(r\hat{\boldsymbol{e}}) + [\nabla_{\boldsymbol{\theta}} U(\boldsymbol{\phi} + r\hat{\boldsymbol{e}}) - \nabla_{\boldsymbol{\theta}} U(r\hat{\boldsymbol{e}})] \Big\} \\
&\geq Mr^2 - \beta - |r| C_g \|\boldsymbol{\phi}\| + 1 - d \quad \text{(by Assumptions 2 and 1)} \\
&\geq Mr^2 - \beta - C_g m_{\boldsymbol{\phi}} B_\varepsilon + 1 - d, \quad \text{(by (A.3) and (A.6))}
\end{aligned}
$$

where the last inequality holds with dominating probability as implied by (A.3) and (A.6)). To conclude the proof, we set

$$\beta_{\boldsymbol{\phi}} := \beta + C_g m_{\boldsymbol{\phi}} B_\varepsilon + d - 1 \asymp C_g. \tag{A.9}$$

□

A.5 Proof of Theorem 4.4

Proof. We provide only the proof for the bound at the times $t = k\eta$ for $k = 1, 2, \ldots$, because it is trivial to generate the results to other times $t \in \mathbb{R}$ by using the same technique as in Raginsky et al. (2017). With a slight abuse of notation, in the proof we reindex π_t and $\hat{\pi}_t$ as π_k and $\hat{\pi}_k$, respectively, to reflect the algorithm's discrete-time updates. Accordingly, we also reindex $\boldsymbol{\theta}_t$ and $\hat{\boldsymbol{\theta}}_t$ as $\boldsymbol{\theta}_k$ and $\hat{\boldsymbol{\theta}}_k$, respectively.

By the definition of the ESGLD sampler,

$$
\begin{aligned}
\mathcal{W}_2^2(\hat{\pi}_{k+1}, \pi_{k+1}) &= \inf_{(\hat{\boldsymbol{\theta}}_{k+1}, \boldsymbol{\theta}_{k+1}) \in \Gamma(\hat{\pi}_{k+1}, \pi_{k+1})} \mathbb{E}\|\hat{\boldsymbol{\theta}}_{k+1} - \boldsymbol{\theta}_{k+1}\|^2 \\
&= \inf_{(\hat{\boldsymbol{\theta}}_{k}, \boldsymbol{\theta}_{k}) \in \Gamma(\hat{\pi}_{k}, \pi_{k})} \mathbb{E}_{\hat{\boldsymbol{\theta}}_k, \boldsymbol{\theta}_k, \boldsymbol{\phi}} \Big\{ \Big(p\mathbb{E}(\|\hat{\boldsymbol{\theta}}_{k+1} - \boldsymbol{\theta}_{k+1}\|^2 | \hat{\boldsymbol{\theta}}_k, \boldsymbol{\theta}_k, mutation \Big) \\
&\quad + (1-p)\mathbb{E}\left(\|\hat{\boldsymbol{\theta}}_{k+1} - \boldsymbol{\theta}_{k+1}\|^2 | \hat{\boldsymbol{\theta}}_k, \boldsymbol{\theta}_k, \boldsymbol{\phi}, snooker) \right) \Big\} \\
&:= p \inf_{(\boldsymbol{\theta}', \boldsymbol{\vartheta}') \in \Gamma(\hat{\pi}_k, \pi_k)} \mathbb{E}(\|Mutation\|^2) + (1-p) \inf_{(\hat{r}_L, r) \in \Gamma(\hat{f}_L, f)} \mathbb{E}(\|Snooker\|^2)
\end{aligned}
$$

where $\Gamma(\cdot, \cdot)$ denotes the coupling of two density functions, $\hat{r}_L$ denotes a sample simulated from $\hat{f}$ by L steps of SGLD moves, $\hat{f}_L$ denotes the distribution of $\hat{r}_L$, and r denotes a sample drawn from f.

Let $t_k = k\eta$. Then

$$
\begin{aligned}
Mutation &= \hat{\boldsymbol{\theta}}_k - \eta\nabla\tilde{U}(\hat{\boldsymbol{\theta}}_k) + \sqrt{2\eta\tau}\boldsymbol{\xi}_{k+1} - \left(\boldsymbol{\theta}_k - \int_{t_k}^{t_{k+1}} \nabla U(\boldsymbol{\theta}_s) ds + \int_{t_k}^{t_{k+1}} \sqrt{2\tau} d\boldsymbol{W}_s \right), \\
Snooker &= \boldsymbol{\phi} + \hat{r}_L \frac{\hat{\boldsymbol{\theta}}_k - \boldsymbol{\phi}}{\|\hat{\boldsymbol{\theta}}_k - \boldsymbol{\phi}\|} - \left(\boldsymbol{\phi} + r\frac{\boldsymbol{\theta}_k - \boldsymbol{\phi}}{\|\boldsymbol{\theta}_k - \boldsymbol{\phi}\|} \right).
\end{aligned}
$$

(i) Mutation Part. By the triangular inequality,

$$
\begin{aligned}
\mathbb{E}\|Mutation\|^2 &= \mathbb{E}\left\| \hat{\boldsymbol{\theta}}_k - \nabla\tilde{U}(\hat{\boldsymbol{\theta}}_k)\eta + \sqrt{2\eta\tau}\boldsymbol{\xi}_{k+1} - \left(\boldsymbol{\theta}_k - \int_{t_k}^{t_{k+1}} \nabla U(\boldsymbol{\theta}_s) ds + \int_{t_k}^{t_{k+1}} \sqrt{2\tau} d\boldsymbol{W}_s \right) \right\|^2 \\
&\le \mathbb{E}\|\hat{\boldsymbol{\theta}}_k - \boldsymbol{\theta}_k\|^2 + 2\underbrace{\mathbb{E}\|\nabla\tilde{U}(\hat{\boldsymbol{\theta}}_k)\eta - \int_{t_k}^{t_{k+1}} \nabla U(\boldsymbol{\theta}_s) ds\|^2}_{(I)} + 2\underbrace{\mathbb{E}\|\sqrt{2\eta\tau}\boldsymbol{\xi}_{k+1} - \sqrt{2\tau}(\boldsymbol{W}_{t_{k+1}} - \boldsymbol{W}_{t_k})\|^2}_{(II)}, \\
&\quad - 2\underbrace{\mathbb{E}\left\langle \hat{\boldsymbol{\theta}}_k - \boldsymbol{\theta}_k, \nabla\tilde{U}(\hat{\boldsymbol{\theta}}_k)\eta - \sqrt{2\eta\tau}\boldsymbol{\xi}_{k+1} - \int_{t_k}^{t_{k+1}} \nabla U(\boldsymbol{\theta}_s) ds + \int_{t_k}^{t_{k+1}} \sqrt{2\tau} d\boldsymbol{W}_s) \right\rangle}_{(III)},
\end{aligned}
$$

where (I) is the term on stochastic gradient noise, (II) is the term on Gaussian white noise, and (III) is the cross-product term. Because $\boldsymbol{\xi}_{k+1}$, $\boldsymbol{W}_s$ (for $s \in (t_k, t_{k+1}]$), and the random noise in $\nabla_{\boldsymbol{\theta}}\tilde{U}(\hat{\boldsymbol{\theta}})$

are all independent of $\hat{\boldsymbol{\theta}}_k - \boldsymbol{\theta}_k$, we have

$$
\begin{aligned}
(III) &= \mathbb{E}\left(\mathbb{E}\left\langle \hat{\boldsymbol{\theta}}_k - \boldsymbol{\theta}_k, \nabla\tilde{U}(\hat{\boldsymbol{\theta}}_k)\eta - \sqrt{2\eta\tau}\boldsymbol{\xi}_{k+1} - \int_{t_k}^{t_{k+1}} \nabla U(\boldsymbol{\theta}_s)ds + \int_{t_k}^{t_{k+1}} \sqrt{2\tau} d\boldsymbol{W}_s)\right\rangle \Big| \hat{\boldsymbol{\theta}}_k, \boldsymbol{\theta}_k\right) \\
&= \mathbb{E}\left(\left\langle \hat{\boldsymbol{\theta}}_k - \boldsymbol{\theta}_k, \nabla U(\hat{\boldsymbol{\theta}}_k)\eta - \int_{t_k}^{t_{k+1}} \nabla U(\boldsymbol{\theta}_s)ds\right\rangle\right) \\
&= \mathbb{E}\left\langle \hat{\boldsymbol{\theta}}_k - \boldsymbol{\theta}_k, \nabla U(\hat{\boldsymbol{\theta}}_k)\eta - \nabla U(\boldsymbol{\theta}_k)\eta + \int_{t_k}^{t_{k+1}} (\nabla U(\boldsymbol{\theta}_k) - \nabla U(\boldsymbol{\theta}_s))ds\right\rangle \\
&= \eta\mathbb{E}\left(\hat{\boldsymbol{\theta}}_k^T \nabla U(\hat{\boldsymbol{\theta}}_k) + \boldsymbol{\theta}_k^T \nabla U(\boldsymbol{\theta}_k) - \boldsymbol{\theta}_k^T \nabla U(\hat{\boldsymbol{\theta}}_k) - \hat{\boldsymbol{\theta}}_k^T \nabla U(\boldsymbol{\theta}_k)\right) \\
&\quad + \underbrace{\mathbb{E}\left((\hat{\boldsymbol{\theta}}_k - \boldsymbol{\theta}_k)^T \int_{t_k}^{t_{k+1}} (\nabla U(\boldsymbol{\theta}_k) - \nabla U(\boldsymbol{\theta}_s))ds\right)}_{(IV)} \\
&\geq \eta\mathbb{E}\left\{M\|\hat{\boldsymbol{\theta}}_k\|^2 + M\|\boldsymbol{\theta}_k\|^2 - 2\beta - \boldsymbol{\theta}_k^T \nabla U(\hat{\boldsymbol{\theta}}_k) - \hat{\boldsymbol{\theta}}_k^T \nabla U(\boldsymbol{\theta}_k)\right\} + (IV).
\end{aligned}
$$

To further bound the term (III), we consider the following results presented in Lemmas 2 and 3 of Raginsky et al. (2017): Suppose Assumptions 1-5 hold, then for all $\boldsymbol{\theta} \in \mathbb{R}^d$,

$$
\|\nabla_{\boldsymbol{\theta}} U(\boldsymbol{\theta})\| \leq C_g\|\boldsymbol{\theta}\| + B; \tag{A.10}
$$

and, if we choose the learning rate $0 < \eta \prec \frac{\sqrt{M}}{4C_g^2}$ such that η satisfies relevant conditions in Lemma A.3 (attainable when M is sufficiently large), then there exists constants c_1 and c_2 such that

$$
\sup_k \mathbb{E}\|\hat{\boldsymbol{\theta}}_k\|^2 \leq c_1, \quad \sup_k \mathbb{E}\|\boldsymbol{\theta}_k\|^2 \leq c_2. \tag{A.11}
$$

Refer to Lemma A.3 for the explicit form of c_1 and c_2. It follows from the assumptions that $B \preceq \sqrt{M}$ and $\beta \prec M$, we have both $c_1 = O(1)$ and $c_2 = O(1)$. Note that we assume the first k-iterations of the ESGLD sampler are performed using SGLD for the warming-up process and, therefore, (A.10) and (A.11) are applicable here. Furthermore, by invoking the Cauchy-Schwarz inequality, we have

$$
\begin{aligned}
&\mathbb{E}\left\{M\|\hat{\boldsymbol{\theta}}_k\|^2 + M\|\boldsymbol{\theta}_k\|^2 - 2\beta - \boldsymbol{\theta}_k^T \nabla U(\hat{\boldsymbol{\theta}}_k) - \hat{\boldsymbol{\theta}}_k^T \nabla U(\boldsymbol{\theta}_k)\right\} \\
&\geq \mathbb{E}\left\{M\|\hat{\boldsymbol{\theta}}_k\|^2 + M\|\boldsymbol{\theta}_k\|^2 - 2\beta - \|\boldsymbol{\theta}_k\|(C_g\|\hat{\boldsymbol{\theta}}_k\| + B) - \|\hat{\boldsymbol{\theta}}_k\|(C_g\|\boldsymbol{\theta}_k\| + B)\right\} \\
&\geq M\mathbb{E}(\|\hat{\boldsymbol{\theta}}_k - \boldsymbol{\theta}_k\|^2) - 2\beta - B(\sqrt{c_1} + \sqrt{c_2}) - \max\{0, C_g - M\}(c_1 + c_2),
\end{aligned} \tag{A.12}
$$

where the last inequality follows from the assumption $\max\{0, C_g - M\} \prec M$ and the upper bound in (A.11).

For term (IV), we first upper bound its absolute value by finding a constant $c_3 \prec M$:

$$
\begin{aligned}
|(IV)| &= \left|\mathbb{E}\left((\hat{\boldsymbol{\theta}}_k - \boldsymbol{\theta}_k)^T \int_{t_k}^{t_{k+1}} (\nabla U(\boldsymbol{\theta}_k) - \nabla U(\boldsymbol{\theta}_s))ds\right)\right| \\
&\leq C_g \int_{t_k}^{t_{k+1}} \left((\mathbb{E}(\|\hat{\boldsymbol{\theta}}_k - \boldsymbol{\theta}_k\|^2))^{1/2}(\mathbb{E}(\|\boldsymbol{\theta}_k - \boldsymbol{\theta}_s\|^2))^{1/2}\right) ds \qquad \text{(A.13)} \\
&\leq C_g\sqrt{2(c_1+c_2)}\left(\sqrt{\frac{C_u}{2}\eta^2} + \frac{4}{3}\sqrt{\tau d}\eta^{3/2}\right) \leq c_3\eta,
\end{aligned}
$$

where C_u is defined in (A.27), the second to the last inequality follows from (A.11) and (A.29), and the existence of c_3 follows from the assumption $\eta \prec \sqrt{M}/(4C_g^2)$. Therefore,

$$
(IV) \geq -c_3\eta. \qquad \text{(A.14)}
$$

As a summary of (A.12) and (A.14), there exists a constant $c_4 := 2\beta + B(\sqrt{c_1} + \sqrt{c_2}) + \max\{0, C_g - M\}(c_1+c_2) + c_3 \prec M$ such that

$$
(III) \geq \eta M \mathbb{E}(\|\hat{\boldsymbol{\theta}}_k - \boldsymbol{\theta}_k\|^2) - c_4\eta.
$$

For the term (I), we have

$$
\begin{aligned}
(I) &\leq 2\mathbb{E}\|\nabla\tilde{U}(\hat{\boldsymbol{\theta}}_k)\eta - \nabla U(\hat{\boldsymbol{\theta}}_k)\eta\|^2 + 2\mathbb{E}\|\nabla U(\hat{\boldsymbol{\theta}}_k)\eta - \int_{t_k}^{t_{k+1}} \nabla U(\boldsymbol{\theta}_s)ds\|^2 \\
&\leq 2\eta^2\mathbb{E}\|\nabla\tilde{U}(\hat{\boldsymbol{\theta}}_k) - \nabla U(\hat{\boldsymbol{\theta}}_k)\|^2 + 4\mathbb{E}\|(\nabla U(\hat{\boldsymbol{\theta}}_k) - \nabla U(\boldsymbol{\theta}_k))\eta\|^2 + 4\mathbb{E}\int_{t_k}^{t_{k+1}} \|\nabla U(\boldsymbol{\theta}_k) - \nabla U(\boldsymbol{\theta}_s)\|^2 ds.
\end{aligned}
$$

By Assumption 2, $\|\nabla U(\boldsymbol{\theta}_1) - \nabla U(\boldsymbol{\theta}_2)\| \leq C_g\|\boldsymbol{\theta}_1 - \boldsymbol{\theta}_2\|$ holds; and by Assumption 4 and (A.11), there exists a constant $\tilde{C} := 2(C_g^2 c_1 + B^2)$ such that

$$
\mathbb{E}\|\nabla\tilde{U}(\hat{\boldsymbol{\theta}}_k) - \nabla U(\hat{\boldsymbol{\theta}}_k)\|^2 \leq \tilde{C}\delta.
$$

Therefore,

$$
(I) \leq 2\tilde{C}\delta\eta^2 + 4\eta^2 C_g^2\mathbb{E}\|\hat{\boldsymbol{\theta}}_k - \boldsymbol{\theta}_k\|^2 + 4C_g^2\int_{t_k}^{t_{k+1}} \mathbb{E}(\|\boldsymbol{\theta}_k - \boldsymbol{\theta}_s\|^2)ds,
$$

where Fubini's theorem has been applied to exchange the order of integration and expectation in the last term of the right. Furthermore, it follows from (A.28) and the condition $\eta \prec \sqrt{M}/(4C_g^2)$, there exists some constant $c_5 \prec M$ such that

$$
4C_g^2\int_{t_k}^{t_{k+1}} \mathbb{E}(\|\boldsymbol{\theta}_k - \boldsymbol{\theta}_s\|^2)ds \leq 4C_g^2(\frac{2}{3}C_u\eta^3 + 2\tau d\eta^2) \prec c_5\eta.
$$

Consequently, we have

$$
(I) \leq 4\eta^2 C_g^2\mathbb{E}(\|\hat{\boldsymbol{\theta}}_k - \boldsymbol{\theta}_k\|^2) + 2\tilde{C}\delta\eta^2 + c_5\eta.
$$

For the term (II), we have

$$(II) \leq 4\eta\tau\mathbb{E}\|\boldsymbol{\xi}_{k+1}\|^2 + 4\tau\mathbb{E}\|(\boldsymbol{W}_{t_{k+1}} - \boldsymbol{W}_{t_k})\|^2 = 8\eta\tau.$$

Summarizing the upper bounds for the terms (I), (II), and (III), we have

$$\mathbb{E}\|mutation\|^2 \leq (1 - 2M\eta + 8C_g^2\eta^2)\mathbb{E}\|\hat{\boldsymbol{\theta}}_k - \boldsymbol{\theta}_k\|^2 + (2c_4 + 2c_5 + 16\tau)\eta + 4\tilde{C}\delta\eta^2. \tag{A.15}$$

(ii) Snooker Part. The derivation for the snooker move is more intricate.

$$\begin{aligned}
\mathbb{E}\|Snooker\|^2 &= \mathbb{E}\left\|\boldsymbol{\phi} + \hat{r}_L\frac{\hat{\boldsymbol{\theta}}_k - \boldsymbol{\phi}}{\|\hat{\boldsymbol{\theta}}_k - \boldsymbol{\phi}\|} - (\boldsymbol{\phi} + r\frac{\boldsymbol{\theta}_k - \boldsymbol{\phi}}{\|\boldsymbol{\theta}_k - \boldsymbol{\phi}\|})\right\|^2 \\
&\leq 2\mathbb{E}\left\|r(\frac{\hat{\boldsymbol{\theta}}_k - \boldsymbol{\phi}}{\|\hat{\boldsymbol{\theta}}_k - \boldsymbol{\phi}\|} - \frac{\boldsymbol{\theta}_k - \boldsymbol{\phi}}{\|\boldsymbol{\theta}_k - \boldsymbol{\phi}\|})\right\|^2 + 2\mathbb{E}\left\|(\hat{r}_L - r)\frac{\hat{\boldsymbol{\theta}}_k - \boldsymbol{\phi}}{\|\hat{\boldsymbol{\theta}}_k - \boldsymbol{\phi}\|}\right\|^2 \\
&= 2\mathbb{E}\left(|r|^2\left\|\frac{\hat{\boldsymbol{\theta}}_k - \boldsymbol{\phi}}{\|\hat{\boldsymbol{\theta}}_k - \boldsymbol{\phi}\|} - \frac{\boldsymbol{\phi} - \boldsymbol{\theta}_k}{\|\boldsymbol{\theta}_k - \boldsymbol{\phi}\|}\right\|^2\right) + 2\mathbb{E}|\hat{r}_L - r|^2
\end{aligned}$$

by observing that $\|\frac{\boldsymbol{\theta}_k - \boldsymbol{\phi}}{\|\boldsymbol{\theta}_k - \boldsymbol{\phi}\|}\| = 1$.

As discussed in Appendix A.2, $\boldsymbol{\phi}$ can be contained in a compact space, uniformly (over all iterations) and with dominating probability. Conditioned on this, for any $\tilde{\varepsilon} > 0$, there exists a small constant $\alpha > 0$ such that $\|\boldsymbol{\theta}_k - \boldsymbol{\phi}\| > \alpha$ and $\|\hat{\boldsymbol{\theta}}_k - \boldsymbol{\phi}\| > \alpha$ hold with probability no smaller than $1 - \tilde{\varepsilon}$, uniformly over all iterations, provided that the population size of the Markov chain and the total number of iterations are both finite. Therefore, the function $\frac{\boldsymbol{\theta} - \boldsymbol{\phi}}{\|\boldsymbol{\theta} - \boldsymbol{\phi}\|}$ is Lipschitz continuous with respect to $\boldsymbol{\theta}$ on $\{\boldsymbol{\theta} : \|\boldsymbol{\theta} - \boldsymbol{\phi}\| > \alpha\}$; there exists a constant c_α such that

$$\left\|\frac{\hat{\boldsymbol{\theta}}_k - \boldsymbol{\phi}}{\|\hat{\boldsymbol{\theta}}_k - \boldsymbol{\phi}\|} - \frac{\boldsymbol{\theta}_k - \boldsymbol{\phi}}{\|\boldsymbol{\theta}_k - \boldsymbol{\phi}\|}\right\| \leq c_\alpha\|\boldsymbol{\theta}_k - \hat{\boldsymbol{\theta}}_k\|. \tag{A.16}$$

As implied by (A.11), we have $c_\alpha = O(1)$.

As discussed previously, the snooker chain also satisfies Assumptions 1-5 with dominating probability. Provided the assumptions are satisfied, by (A.11), we can find a constant $c_6 = O(1)$ such that $\mathbb{E}(|r|^2|\boldsymbol{\theta}_k, \boldsymbol{\phi}) \leq c_6$. In this case,

$$\begin{aligned}
\inf_{\Gamma(\hat{f}_L, f)} \mathbb{E}\|Snooker\|^2 &\leq 2c_\alpha^2 c_6 \inf_{\Gamma(\hat{\pi}_k, \pi_k)} \mathbb{E}(\|\boldsymbol{\theta}_k - \hat{\boldsymbol{\theta}}_k\|^2) + 2\inf_{\Gamma(\hat{f}_L, f)} \mathbb{E}|\hat{r}_L - r|^2 \\
&= 2c_\alpha^2 c_6 \inf_{\Gamma(\hat{\pi}_k, \pi_k)} \mathbb{E}(\|\boldsymbol{\theta}_k - \hat{\boldsymbol{\theta}}_k\|^2) + 2\mathcal{W}_2^2(\hat{r}_L, r),
\end{aligned}$$

where the inequality follows from the fact that r and $\boldsymbol{\theta}_k - \hat{\boldsymbol{\theta}}_k$ are conditionally independent given $\boldsymbol{\theta}_k$. For the term $\mathcal{W}_2^2(\hat{r}_L, r)$, by the triangular inequality of 2-Wasserstein distance, we have

$$\mathcal{W}_2^2(\hat{r}_L, r) \leq 2\mathcal{W}_2^2(\hat{r}_L, \hat{r}) + 2\mathcal{W}_2^2(\hat{r}, r), \tag{A.17}$$

where $\hat{r}$ has a density function given by $\hat{f}(r) \propto |r|^{d-1}\pi(\boldsymbol{\phi} + r\frac{\hat{\boldsymbol{\theta}}_k - \boldsymbol{\phi}}{\|\hat{\boldsymbol{\theta}}_k - \boldsymbol{\phi}\|})$.

Under Assumptions 1-5, Raginsky et al. (2017) has established the $\mathcal{W}_2$ distance between the distribution of a SGLD sample and its target distribution. By the result of Raginsky et al. (2017), for the snooker chain, we have

$$\mathcal{W}_2^2(\hat{r}_L, \hat{r}) \le D_2(\tau, d, M, \beta_{\boldsymbol{\phi}}, C_{\boldsymbol{\phi}}, \eta_r, \delta, L), \tag{A.18}$$

where η_r is the step size used in sampling $\hat{r}_L$. By Proposition 10 of Raginsky et al. (2017),

$$D_2 \le c_{7,1}\delta^{\frac{1}{2}} L^2 \eta_r^2 + c_{7,2} L^2 \eta_r^{\frac{5}{2}} + c_{7,3} e^{-2L\eta_r\tau/c_{LS}}, \tag{A.19}$$

for some constants $c_{7,1} > 0$, $c_{7,2} > 0$, $c_{7,3} > 0$, $c_7 > 0$, and $c_{LS} > 0$, if we set $\eta_r \le \eta \prec \sqrt{M}/(4C_g^2)$, and L is sufficiently large. Here c_{LS} denotes the logarithmic Sobolev constant of the distribution $\hat{f}(r)$. By the derivations in (A.8) and (A.9) and Proposition 10 in Raginsky et al. (2017), we have $c_{7,1} = O(C_g^3)$, $c_{7,2} = O(C_g^5)$, and $C_{7,3} = O(C_g)$. Note that our energy function setting and Assumptions 1-3 imply $C_g = O(M)$, $B^2 \preceq C_g$, and $A \preceq C_g$. Regarding η_r and L, we may set $0 < \eta_r \le \eta \prec \sqrt{M}/(4C_g^2)$ and $L = -\frac{s\log(\eta_r)}{\eta_r}$ for some constant $s > c_{LS}/(4\tau)$ such that $c_{7,3}e^{-2L\eta_r\tau/c_{LS}} = o(1)$ holds. For example, we can set $\eta_r = \eta$, $L = -s\log(\eta)/\eta$, and $s = c_{LS}/(2\tau)$. Consequently, we can find a constant $c_7 = O(1)$ such that

$$D_2 \le c_7 \log^2(\eta)(C_g^3\delta^{\frac{1}{2}} + C_g^5\eta^{\frac{1}{2}}). \tag{A.20}$$

Now let's work on $\mathcal{W}_2^2(\hat{r}, r)$, squared 2-Wasserstein distance between $\hat{f}$ and f, where $\hat{f}(\hat{r}) \propto |\hat{r}|^{d-1}\pi(\boldsymbol{\phi} + \hat{r}\frac{\hat{\boldsymbol{\theta}}_k - \boldsymbol{\phi}}{\|\hat{\boldsymbol{\theta}}_k - \boldsymbol{\phi}\|})$ and $f(r) \propto |r|^{d-1}\pi(\boldsymbol{\phi} + r\frac{\boldsymbol{\theta}_k - \boldsymbol{\phi}}{\|\boldsymbol{\theta}_k - \boldsymbol{\phi}\|})$. By noting that $\hat{r}$ and r are conditionally independent given $\boldsymbol{\theta}_k$, $\hat{\boldsymbol{\theta}}_k$ and $\boldsymbol{\phi}$, we have

$$\begin{aligned} &\mathbb{E}(|\hat{r} - r|^2 | \boldsymbol{\theta}_k, \hat{\boldsymbol{\theta}}_k, \boldsymbol{\phi}) = \mathbb{E}_{\boldsymbol{\theta}_k, \hat{\boldsymbol{\theta}}_k, \boldsymbol{\phi}}\left(\int (r - \hat{r})^2 \hat{f}(\hat{r}) f(r) d\hat{r} dr\right) \\ &= \mathbb{E}_{\boldsymbol{\theta}_k, \hat{\boldsymbol{\theta}}_k, \boldsymbol{\phi}}\left(\int (r - \hat{r})^2 |\hat{r}|^{d-1}\pi(\boldsymbol{\phi} + \hat{r}\frac{\hat{\boldsymbol{\theta}}_k - \boldsymbol{\phi}}{\|\hat{\boldsymbol{\theta}}_k - \boldsymbol{\phi}\|})|r|^{d-1}\pi(\boldsymbol{\phi} + r\frac{\boldsymbol{\theta}_k - \boldsymbol{\phi}}{\|\boldsymbol{\theta}_k - \boldsymbol{\phi}\|}) d\hat{r} dr\right), \end{aligned} \tag{A.21}$$

where $\mathbb{E}_{\boldsymbol{\theta}_k, \hat{\boldsymbol{\theta}}_k, \boldsymbol{\phi}}(\cdot)$ denotes taking expectation with respect to the conditional distribution of $(r, \hat{r})$ given $(\boldsymbol{\theta}_k, \hat{\boldsymbol{\theta}}_k, \boldsymbol{\phi})$.

By Proposition 1, π is continuously differentiable. By the Lagrangian mean value theorem (LMVT), for any r, there exists $\lambda \in [0, 1]$ such that $\boldsymbol{\zeta} = \lambda\frac{\boldsymbol{\theta}_k - \boldsymbol{\phi}}{\|\boldsymbol{\theta}_k - \boldsymbol{\phi}\|} + (1 - \lambda)\frac{\hat{\boldsymbol{\theta}}_k - \boldsymbol{\phi}}{\|\hat{\boldsymbol{\theta}}_k - \boldsymbol{\phi}\|}$ and the following holds:

$$\begin{aligned} \pi(\boldsymbol{\phi} + r\frac{\boldsymbol{\theta}_k - \boldsymbol{\phi}}{\|\boldsymbol{\theta}_k - \boldsymbol{\phi}\|}) - \pi(\boldsymbol{\phi} + r\frac{\hat{\boldsymbol{\theta}}_k - \boldsymbol{\phi}}{\|\hat{\boldsymbol{\theta}}_k - \boldsymbol{\phi}\|})) &= \langle \nabla_{\boldsymbol{\zeta}}\pi(\boldsymbol{\phi} + r\boldsymbol{\zeta}), \frac{\boldsymbol{\theta}_k - \boldsymbol{\phi}}{\|\boldsymbol{\theta}_k - \boldsymbol{\phi}\|} - \frac{\hat{\boldsymbol{\theta}}_k - \boldsymbol{\phi}}{\|\hat{\boldsymbol{\theta}}_k - \boldsymbol{\phi}\|}\rangle \\ &\le \|\nabla_{\boldsymbol{\zeta}}\pi(\boldsymbol{\phi} + r\boldsymbol{\zeta})\| \left\| \frac{\boldsymbol{\theta}_k - \boldsymbol{\phi}}{\|\boldsymbol{\theta}_k - \boldsymbol{\phi}\|} - \frac{\hat{\boldsymbol{\theta}}_k - \boldsymbol{\phi}}{\|\hat{\boldsymbol{\theta}}_k - \boldsymbol{\phi}\|} \right\|, \end{aligned}$$

where the inequality follows from the Cauchy-Schwartz inequality. Note that

$$\|\nabla_{\boldsymbol{\zeta}}\pi(\boldsymbol{\phi}+r\boldsymbol{\zeta})\|=|r|\|\nabla_{\boldsymbol{\phi}+r\boldsymbol{\zeta}}\pi(\boldsymbol{\phi}+r\boldsymbol{\zeta})\|,$$

and that, by (A.10), $\|\nabla_{\boldsymbol{\theta}}U(\boldsymbol{\theta})\|=\frac{\|\nabla_{\boldsymbol{\theta}}\pi(\boldsymbol{\theta})\|}{\pi(\boldsymbol{\theta})}<C_g\|\boldsymbol{\theta}\|+B$. Furthermore, by (A.16) and (A.6), we have

$$\begin{aligned}\pi(\boldsymbol{\phi}+r\frac{\boldsymbol{\theta}_k-\boldsymbol{\phi}}{\|\boldsymbol{\theta}_k-\boldsymbol{\phi}\|})-\pi(\boldsymbol{\phi}+r\frac{\hat{\boldsymbol{\theta}}_k-\boldsymbol{\phi}}{\|\hat{\boldsymbol{\theta}}_k-\boldsymbol{\phi}\|})) &\leq |r|\|\nabla_{\boldsymbol{\phi}+r\boldsymbol{\zeta}}\pi(\boldsymbol{\phi}+r\boldsymbol{\zeta})\|c_\alpha\|\hat{\boldsymbol{\theta}}_k-\boldsymbol{\theta}_k\|\\ &\leq |r|(C_g\|\boldsymbol{\phi}+r\zeta\|+B)\pi(\boldsymbol{\phi}+r\boldsymbol{\zeta})c_\alpha\|\hat{\boldsymbol{\theta}}_k-\boldsymbol{\theta}_k\|\\ &\leq c_\alpha(a_1|r|^2+b_1|r|)\pi(\boldsymbol{\phi}+r\boldsymbol{\zeta})\|\hat{\boldsymbol{\theta}}_k-\boldsymbol{\theta}_k\|,\end{aligned}$$

for some constants a_1 and b_1. Back to equation (A.21), we thus have

$$\begin{aligned}&\mathbb{E}(|\hat{r}-r|^2|\boldsymbol{\theta}_k,\hat{\boldsymbol{\theta}}_k,\boldsymbol{\phi})\leq \mathbb{E}_{\boldsymbol{\theta}_k,\hat{\boldsymbol{\theta}}_k,\boldsymbol{\phi}}\Big[\int(r-\hat{r})^2|\hat{r}|^{d-1}\pi(\boldsymbol{\phi}+\hat{r}\frac{\hat{\boldsymbol{\theta}}_k-\boldsymbol{\phi}}{\|\hat{\boldsymbol{\theta}}_k-\boldsymbol{\phi}\|})\\ &\quad\times|r|^{d-1}\left(\pi(\boldsymbol{\phi}+r\frac{\hat{\boldsymbol{\theta}}_k-\boldsymbol{\phi}}{\|\hat{\boldsymbol{\theta}}_k-\boldsymbol{\phi}\|})+c_\alpha(a_1|r|^2+b_1|r|)\|\hat{\boldsymbol{\theta}}_k-\boldsymbol{\theta}_k\|\pi(\boldsymbol{\phi}+r\zeta)\right)d\hat{r}dr\Big]\\ &=\mathbb{E}_{\boldsymbol{\theta}_k,\hat{\boldsymbol{\theta}}_k,\boldsymbol{\phi}}\Big[\int(r-\hat{r})^2|\hat{r}|^{d-1}\pi(\boldsymbol{\phi}+\hat{r}\frac{\hat{\boldsymbol{\theta}}_k-\boldsymbol{\phi}}{\|\hat{\boldsymbol{\theta}}_k-\boldsymbol{\phi}\|})|r|^{d-1}\pi(\boldsymbol{\phi}+r\frac{\hat{\boldsymbol{\theta}}_k-\boldsymbol{\phi}}{\|\hat{\boldsymbol{\theta}}_k-\boldsymbol{\phi}\|})d\hat{r}dr\Big]\\ &\quad+c_\alpha\mathbb{E}_{\boldsymbol{\theta}_k,\hat{\boldsymbol{\theta}}_k,\boldsymbol{\phi}}\Big[\|\hat{\boldsymbol{\theta}}_k-\boldsymbol{\theta}_k\|\int(r-\hat{r})^2(a_1|r|^2+b_1|r|)|\hat{r}|^{d-1}\pi(\boldsymbol{\phi}+\hat{r}\frac{\hat{\boldsymbol{\theta}}_k-\boldsymbol{\phi}}{\|\hat{\boldsymbol{\theta}}_k-\boldsymbol{\phi}\|})|r|^{d-1}\pi(\boldsymbol{\phi}+r\zeta)d\hat{r}dr\Big]\end{aligned}$$

where the first term of the right, upon taking infimum by setting $\hat{r}=r$, is just $\mathcal{W}_2(f,f)=0$. We can further apply LMVT to the second term, and define $\boldsymbol{\zeta}_2=\lambda_2\boldsymbol{\zeta}+(1-\lambda_2)\frac{\hat{\boldsymbol{\theta}}_k-\boldsymbol{\phi}}{\|\hat{\boldsymbol{\theta}}_k-\boldsymbol{\phi}\|}$. With a similar derivation, we can find some constants a_2 and b_2 such that

$$\begin{aligned}&\int(r-\hat{r})^2(a_1|r|^2+b_1|r|)|\hat{r}|^{d-1}\pi(\boldsymbol{\phi}+\hat{r}\frac{\hat{\boldsymbol{\theta}}_k-\boldsymbol{\phi}}{\|\hat{\boldsymbol{\theta}}_k-\boldsymbol{\phi}\|})|r|^{d-1}\pi(\boldsymbol{\phi}+r\boldsymbol{\zeta})d\hat{r}dr\\ &\leq\int(r-\hat{r})^2(a_1|r|^2+b_1|r|)|\hat{r}|^{d-1}\pi(\boldsymbol{\phi}+\hat{r}\boldsymbol{\zeta})|r|^{d-1}\pi(\boldsymbol{\phi}+r\boldsymbol{\zeta})d\hat{r}dr\\ &+c_\alpha\lambda\|\hat{\boldsymbol{\theta}}_k-\boldsymbol{\theta}_k\|\int(r-\hat{r})^2(a_1|r|^2+b_1|r|)(a_2|\hat{r}|^2+b_2|\hat{r}|)|\hat{r}|^{d-1}\pi(\boldsymbol{\phi}+\hat{r}\boldsymbol{\zeta}_2)|r|^{d-1}\pi(\boldsymbol{\phi}+r\boldsymbol{\zeta})d\hat{r}dr,\end{aligned}$$

where the first term of the right, again by choosing $\hat{r}=r$, becomes 0. The factor $c_\alpha\lambda\|\hat{\boldsymbol{\theta}}_k-\boldsymbol{\theta}_k\|$ in the second term comes from $\|\boldsymbol{\zeta}-\frac{\hat{\boldsymbol{\theta}}_k-\boldsymbol{\phi}}{\|\hat{\boldsymbol{\theta}}_k-\boldsymbol{\phi}\|}\|=\|\lambda\frac{\boldsymbol{\theta}_k-\boldsymbol{\phi}}{\|\boldsymbol{\theta}_k-\boldsymbol{\phi}\|}+(1-\lambda)\frac{\hat{\boldsymbol{\theta}}_k-\boldsymbol{\phi}}{\|\hat{\boldsymbol{\theta}}_k-\boldsymbol{\phi}\|}-\frac{\hat{\boldsymbol{\theta}}_k-\boldsymbol{\phi}}{\|\hat{\boldsymbol{\theta}}_k-\boldsymbol{\phi}\|}\|=\lambda\|\frac{\boldsymbol{\theta}_k-\boldsymbol{\phi}}{\|\boldsymbol{\theta}_k-\boldsymbol{\phi}\|}-\frac{\hat{\boldsymbol{\theta}}_k-\boldsymbol{\phi}}{\|\hat{\boldsymbol{\theta}}_k-\boldsymbol{\phi}\|}\|\leq c_\alpha\lambda\|\hat{\boldsymbol{\theta}}_k-\boldsymbol{\theta}_k\|$. Hence, there exists a constant $c_8>0$ such that

$$\begin{aligned}\mathbb{E}(|\hat{r}-r|^2|\boldsymbol{\theta}_k,\hat{\boldsymbol{\theta}}_k,\boldsymbol{\phi})&\leq\lambda c_\alpha^2\mathbb{E}_{\boldsymbol{\theta}_k,\hat{\boldsymbol{\theta}}_k,\boldsymbol{\phi}}\Big(\|\hat{\boldsymbol{\theta}}_k-\boldsymbol{\theta}_k\|^2\int(r-\hat{r})^2(a_1|r|^2+b_1|r|)(a_2|\hat{r}|^2+b_2|\hat{r}|)\\ &\qquad\times|\hat{r}|^{d-1}\pi(\boldsymbol{\phi}+\hat{r}\boldsymbol{\zeta}_2)|r|^{d-1}\pi(\boldsymbol{\phi}+r\boldsymbol{\zeta})d\hat{r}dr\Big)\\ &\leq\lambda c_\alpha^2c_8\|\hat{\boldsymbol{\theta}}_k-\boldsymbol{\theta}_k\|^2,\end{aligned}$$

where the second inequality follows from (A.2), and $c_8 = O(1)$ as implied by (A.11). Specifically, (A.2) implies the following holds with dominating probability:

$$\int (r-\hat{r})^2(a_1|r|^2+b_1|r|)(a_2|\hat{r}|^2+b_2|\hat{r}|)|\hat{r}|^{d-1}\pi(\boldsymbol{\phi}+\hat{r}\boldsymbol{\zeta}_2)|r|^{d-1}\pi(\boldsymbol{\phi}+r\boldsymbol{\zeta})d\hat{r}dr \le c_8.$$

In summary, we have shown that the following holds with dominating probability:

$$\inf_{\Gamma(\hat{f}_L,f)} \mathbb{E}\|Snooker\|^2 \le 4c_7\log^2(\eta)(C_g^3\delta^{\frac{1}{2}} + C_g^5\eta^{\frac{1}{2}}) + 2c_\alpha^2(c_6+2\lambda c_8)\inf_{\Gamma(\hat{\pi}_k,\pi_k)} \mathbb{E}\left(\|\hat{\boldsymbol{\theta}}_k - \boldsymbol{\theta}_k\|^2\right). \tag{A.22}$$

Combining the mutation and snooker parts, the following holds with dominating probability:

$$\begin{aligned}
\mathcal{W}_2^2(\hat{\pi}_{k+1},\pi_{k+1}) &\le \inf_{\Gamma(\hat{\pi}_k,\pi_k)} p\left(\mathbb{E}\|mutation\|^2\right) + (1-p)\inf_{\Gamma(\hat{f}_L,f)}\left(\mathbb{E}\|Snooker\|^2\right)\\
&\le p\left((1-2M\eta+8C_g^2\eta^2)\inf_{\Gamma(\hat{\pi}_k,\pi_k)}\mathbb{E}\|\hat{\boldsymbol{\theta}}_k-\boldsymbol{\theta}_k\|^2+(2c_4+2c_5+16\tau)\eta+4\tilde{C}\delta\eta^2\right)\\
&\quad + (1-p)\left(4c_7\log^2(\eta)(C_g^3\delta^{\frac{1}{2}}+C_g^5\eta^{\frac{1}{2}}) + 2c_\alpha^2(c_6+2\lambda c_8)\inf_{\Gamma(\hat{\pi}_k,\pi_k)}\mathbb{E}\|\hat{\boldsymbol{\theta}}_k-\boldsymbol{\theta}_k\|^2\right)\\
&= \left(p(1-2M\eta+8C_g^2\eta^2)+(1-p)2c_\alpha^2(c_6+2\lambda c_8)\right)\mathcal{W}_2^2(\hat{\pi}_k,\pi_k)\\
&\quad + 4(1-p)c_7\log^2(\eta)(C_g^3\delta^{\frac{1}{2}}+C_g^5\eta^{\frac{1}{2}}) + 2p(c_4+c_5+8\tau)\eta + 4p\tilde{C}\delta\eta^2.
\end{aligned} \tag{A.23}$$

Further, if we set $\omega = 2c_\alpha^2(c_6+2\lambda c_8) - 1 > 0$ by choosing c_6 and c_8 appropriately, and set

$$p > \frac{\omega}{\omega + 2M\eta - 8C_g^2\eta^2},$$

then $\varrho := p(1-2M\eta+8C_g^2\eta^2) + (1-p)(\omega+1) < 1$ and, consequently,

$$\mathcal{W}_2^2(\hat{\pi}_{k+1},\pi_{k+1}) \le \varrho\mathcal{W}_2^2(\hat{\pi}_k,\pi_k) + 4(1-p)c_7\log^2(\eta)(C_g^3\delta^{\frac{1}{2}}+C_g^5\eta^{\frac{1}{2}}) + 2p(c_4+c_5+8\tau)\eta + 4p\tilde{C}\delta\eta^2. \tag{A.24}$$

For the next iteration, i.e., iteration $k+2$, we cannot assume that only SGLD moves are performed during the first $k+1$ iterations. In this case, the values of c_1 and c_2 need to be changed (and thus, the values of c_3, c_4, and c_5 need to be adjusted accordingly). However, since the snooker move is executed with SGLD, $\mathbb{E}\|\hat{\boldsymbol{\theta}}_{k+1}\|^2$ and $\mathbb{E}\|\boldsymbol{\theta}_{k+1}\|^2$ are upper-bounded. Therefore, with appropriately chosen constants c_1 and c_2, (A.11) can hold for iteration $k+1$ and all subsequent iterations. In particular, (A.6) holds uniformly over all iterations of the ESGLD sampler due to the underlying dissipativity condition, as discussed in Section A.2. Therefore, (A.24) holds for iteration $k+2$ and all subsequent iterations. This concludes the proof of the recursion (9).

To show that $\mathcal{W}_2^2(\hat{\pi}_{k+1},\pi_{k+1}) \to 0$ as $k\to\infty$, $\eta\to 0$, and $N\to\infty$, we proceed as follows. For any $0<\eta<1$, we can find some constants $c_0 \ge 1$, $1<\psi<2$ and $0<\upsilon<1$ such that

$\eta^{\upsilon} = 2c_0\eta - \eta^{\psi}$. If we set $p = \frac{\omega}{\omega + M\eta^{\psi}/c_0 - 8C_g^2\eta^2}$, then

$$\varrho = 1 - \frac{\eta^{\upsilon}\omega M/c_0}{\omega + M\eta^{\psi}/c_0 - 8C_g^2\eta^2}. \tag{A.25}$$

and thus

$$\lim_{k\to\infty} \mathcal{W}_2^2(\hat{\pi}_k, \pi_k) \le \frac{4c_7\log^2(\eta)}{\omega}\eta^{\psi-\upsilon}(C_g^3\delta^{\frac{1}{2}} + C_g^5\eta^{\frac{1}{2}}) + \frac{2c_0(c_4 + c_5 + 8\tau)}{M}\eta^{1-\upsilon} + \frac{4c_0\tilde{C}\delta}{M}\eta^{2-\upsilon}.$$

Letting $\eta \to 0$, we observe that $\upsilon \to 1$. It is straightforward to show that if we set $\eta \prec 1/C_g^{\varsigma}$ and choose

$$\psi \in \left(1 + \frac{3}{\varsigma},\ 2 - \frac{1}{\varsigma}\right) \quad \text{with} \quad \varsigma > 4,$$

then the condition $0 < p < 1$ is satisfied, and we obtain

$$\frac{4c_7\log^2(\eta)}{\omega}\eta^{\psi-\upsilon}\left(C_g^3\delta^{1/2} + C_g^5\eta^{1/2}\right) = o(1).$$

Furthermore, by noting $c_4 \prec M$, $c_5 \prec M$, $M = O(N^{\alpha})$, $\tau = O(1)$, and $\tilde{C} = O(C_g^2)$,

$$\lim_{k\to\infty} \mathcal{W}_2^2(\hat{\pi}_k, \pi_k) \le o(1) + \frac{2c_0(c_4 + c_5 + 8\tau)}{M} + o(\frac{1}{MC_g^2}) = o(1),$$

as $\eta \to 0$ and $N \to \infty$. In summary, we have

$$\lim_{N\to\infty}\lim_{\eta\to 0}\lim_{k\to\infty} \mathcal{W}_2^2(\hat{\pi}_k, \pi_k) = 0. \tag{A.26}$$

□

A.6 On the order of $\int_{t_k}^{t_{k+1}} \mathbb{E}\|\boldsymbol{\theta}_k - \boldsymbol{\theta}_s\|^2\, ds$

Let $\boldsymbol{\theta}_s$ evolve according to the overdamped Langevin SDE:

$$d\boldsymbol{\theta}_s = -\nabla U(\boldsymbol{\theta}_s)\, ds + \sqrt{2\tau}\, dW_s,$$

with $\boldsymbol{\theta}_{t_k} = \boldsymbol{\theta}_k$. Then,

$$\boldsymbol{\theta}_s = \boldsymbol{\theta}_k - \int_{t_k}^{s} \nabla U(\boldsymbol{\theta}_r)\, dr + \sqrt{2\tau}(W_s - W_{t_k}).$$

Hence,

$$\boldsymbol{\theta}_s - \boldsymbol{\theta}_k = -\int_{t_k}^{s} \nabla U(\boldsymbol{\theta}_r)\, dr + \sqrt{2\tau}(W_s - W_{t_k}).$$

Taking the squared norm and using the inequality $\|a + b\|^2 \le 2\|a\|^2 + 2\|b\|^2$, we have

$$\|\boldsymbol{\theta}_s - \boldsymbol{\theta}_k\|^2 \le 2\left\|\int_{t_k}^{s} \nabla U(\boldsymbol{\theta}_r)\, dr\right\|^2 + 4\tau\|W_s - W_{t_k}\|^2.$$

Taking expectation on both sides:

$$\mathbb{E}\|\boldsymbol{\theta}_s - \boldsymbol{\theta}_k\|^2 \leq 2\,\mathbb{E}\left\|\int_{t_k}^{s} \nabla U(\boldsymbol{\theta}_r)\,dr\right\|^2 + 4\tau\,\mathbb{E}\|W_s - W_{t_k}\|^2.$$

It follows from (A.10) that

$$\|\nabla U(\boldsymbol{\theta})\|^2 \leq 2C_g^2\|\boldsymbol{\theta}\|^2 + 2B^2,$$

and it follows from (A.11) that $\sup_{r\in[t_k,t_{k+1}]}\mathbb{E}\|\boldsymbol{\theta}_r\|^2 \leq c_2$. Then,

$$\mathbb{E}\|\nabla U(\boldsymbol{\theta}_r)\|^2 \leq 2C_g^2 c_2 + 2B^2 := C_u. \tag{A.27}$$

By the Cauchy-Schwarz inequality,

$$\mathbb{E}\left\|\int_{t_k}^{s} \nabla U(\boldsymbol{\theta}_r)\,dr\right\|^2 \leq (s - t_k)\int_{t_k}^{s} \mathbb{E}\|\nabla U(\boldsymbol{\theta}_r)\|^2 dr \leq (s - t_k)^2 C_u.$$

Also, since $W_s - W_{t_k} \sim \mathcal{N}(0, (s - t_k)I)$, we have

$$\mathbb{E}\|W_s - W_{t_k}\|^2 = d(s - t_k),$$

where d is the dimension of $\boldsymbol{\theta}$. Thus,

$$\mathbb{E}\|\boldsymbol{\theta}_s - \boldsymbol{\theta}_k\|^2 \leq 2C_u(s - t_k)^2 + 4\tau d(s - t_k).$$

Integrate over $s \in [t_k, t_{k+1}]$, where $\eta = t_{k+1} - t_k$:

$$\int_{t_k}^{t_{k+1}} \mathbb{E}\|\boldsymbol{\theta}_s - \boldsymbol{\theta}_k\|^2\,ds \leq \int_0^{\eta} \left(2C_u s^2 + 4\tau d s\right) ds = \frac{2}{3}C_u\eta^3 + 2\tau d\eta^2 = O(C_g^2\eta^3 + \tau d\eta^2). \tag{A.28}$$

Additionally,

$$\begin{aligned}\int_{t_k}^{t_{k+1}} \sqrt{\mathbb{E}\|\boldsymbol{\theta}_s - \boldsymbol{\theta}_k\|^2}\,ds &\leq \int_0^{\eta} \left(\sqrt{2C_u}s + 2\sqrt{\tau d}s^{1/2}\right) ds = \sqrt{\frac{C_u}{2}}\eta^2 + \frac{4}{3}\sqrt{\tau d}\eta^{\frac{3}{2}} \\ &= O(C_g\eta^2 + \sqrt{\tau d}\eta^{\frac{3}{2}}).\end{aligned} \tag{A.29}$$

A.7 Proof of Corollary 4.5

Lemma A.4. *(Polyanskiy and Wu (2016), Raginsky et al. (2017)) Let μ and ν be two probability distributions on $\mathbb{R}^d$ with finite second moments, and let $h : \mathbb{R}^d \to \mathbb{R}$ be a continuously differentiable function satisfying $\|\nabla h(\boldsymbol{x})\| \leq c_1\|\boldsymbol{x}\| + c_2$ for some constants $c_1 > 0$ and $c_2 \geq 0$. Then*

$$\left|\int_{\mathbb{R}^d} h d\mu - \int_{\mathbb{R}^d} h d\nu\right| \leq c_3\mathcal{W}_2(\mu, \nu),$$

where $c_3 = (c_1\sigma + c_2)$ and $\sigma = \sqrt{\max\{\int_{\mathbb{R}^d}\|\boldsymbol{x}\|^2 d\mu, \int_{\mathbb{R}^d}\|\boldsymbol{x}\|^2 d\nu\}}$.

Proof of Corollary 4.5

Proof. It follows directly from Theorem 4.4 and Lemma A.4. □

B Numerical Experiments

B.1 A Mixture Gaussian Distribution

Table A1 provides the centers of the 20-component mixture Gaussian distribution.

Table A1: Centers of the 20-component mixture Gaussian distribution

i	$\mu_{i,1}$	$\mu_{i,2}$	i	$\mu_{i,1}$	$\mu_{i,2}$
1	2.547309	2.547301	11	9.616817	6.089943
2	1.005194	1.910528	12	1.475194	9.411089
3	6.898408	8.405147	13	1.15205	1.08722
4	9.168438	5.954380	14	8.724225	4.368250
5	7.154848	3.854593	15	3.652639	8.852135
6	9.028120	9.139374	16	8.746123	2.893911
7	2.140865	8.745735	17	7.246912	2.387444
8	6.635207	7.449630	18	4.365018	6.759031
9	9.126848	0.917082	19	6.467183	6.694622
10	0.033064	5.541101	20	2.876139	6.853250

Experimental Setup: For this example, the ESGLD algorithm was executed with a population of 20 chains for 30,000 iterations. The chains are initialized uniformly in $[0, 10] \times [0, 10]$ square (the chains are initialized uniformly in $[0, 1] \times [0, 1]$ in the main text). The temperature ladder was equi-spaced from 1.0 to 10.0, with the corresponding learning rates equi-spaced from 0.001 to 0.021. The selection temperature was set to 0.5, and the mutation rate was set to 0.7. The snooker operator runs for 10 iterations. The total CPU time for a single run was approximately 95 CPU seconds on a computer with a 2.6 GHz processor. For comparison, other algorithms were performed at a temperature of 1 and learning rate 0.001 on the same computer with the same CPU cost, except for cyclical SGLD which has a periodic cosine decay in the step size that motivated us to set the learning rate at 0.002. For Parallel Tempering (PT) and Evolutionary Monte Carlo (EMC), we use the Metropolis-Hasting algorithm with the random walk proposal $\mathcal{N}(\boldsymbol{\theta}, I_2)$ for local updates at each

temperature level. For the snooker operator in EMC, we use the random walk proposal $\mathcal{N}(r, 1)$ in the Metropolis-Hastings update. Each algorithm's sample path was appropriately thinned to yield a sample size of 30,000. Since ESGLD is designed to be scalable, we additionally compare it with gradient-based algorithms, including SGLD, SGHMC, cSGLD, RESGLD, pSGLD, MSGLD, and SGNHT, in what follows.

B.1.1 Sensitivity to Learning Rates

Considering that the learning rate 0.001 is very small, we conducted the experiments again, but with a larger learning rate of 0.01 this time. Figure 1 and Figure 2 show the scatter plots and sample paths of the first 4/15 iterations produced by different algorithms. Table A2 compares the estimates of the mean vector and covariance matrix of the mixture distribution.

With the increased learning rate, ESGLD and RESGLD are able to visit all modes and accurately estimates the mean and covariance matrix. With the exception of MSGLD, none of the other algorithms were able to visit all modes. However, MSGLD overestimates both $\mathrm{Var}(\mathrm{X}_1)$ and $\mathrm{Var}(\mathrm{X}_2)$.

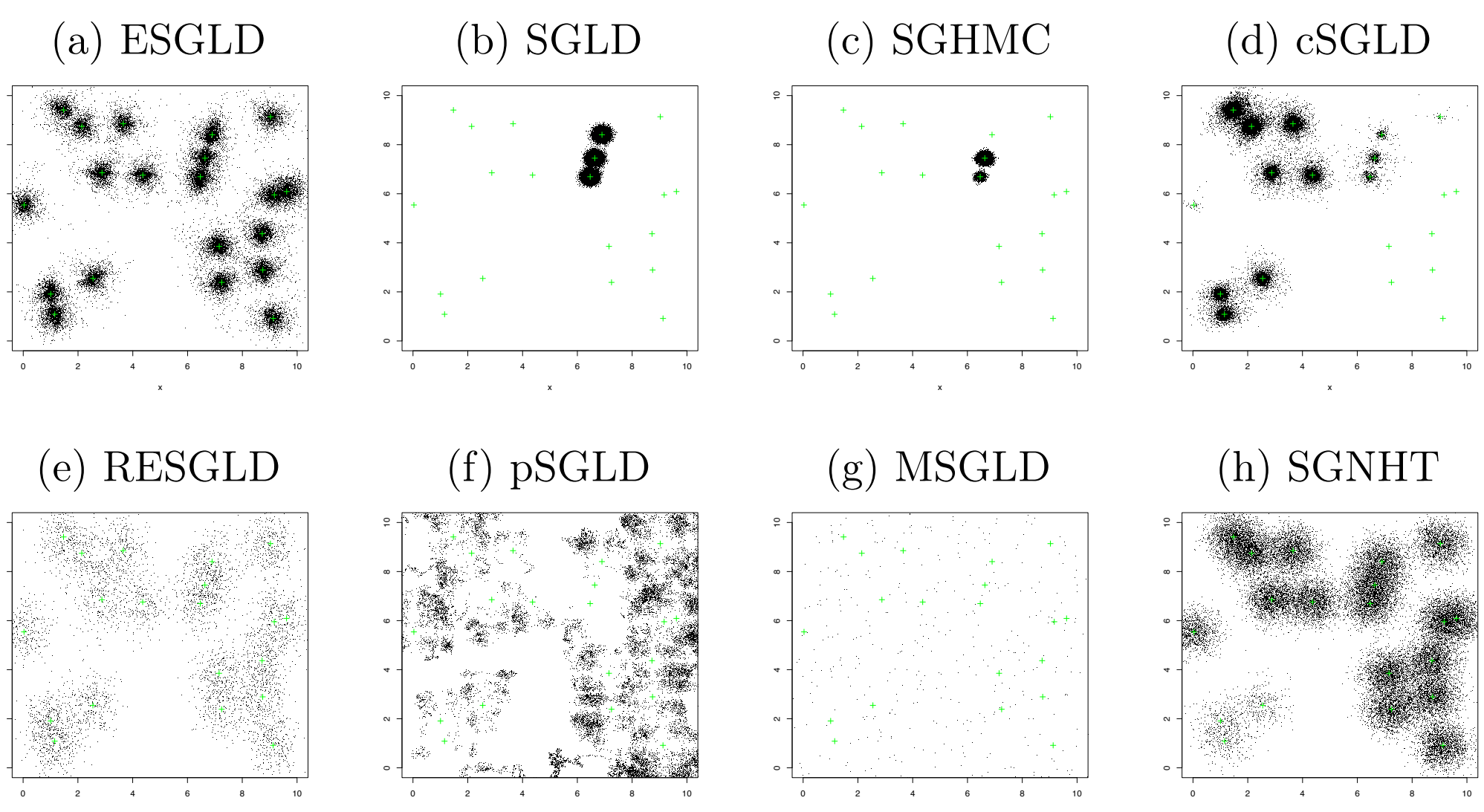

Figure 1: scatter plots of the samples produced by different algorithms with a learning rate of 0.01.

B.1.2 Sensitivity to Population Size, Temperature Ladder, and Mutation Rates

We further explored different experimental setups for ESGLD. In the first set of experiments, we let the population size (i.e., number of chains) be 5 and the learning rate be equally spaced from 0.001 to 0.021. We conducted the experiments under three scenarios with the temperature ladders

(a) ESGLD (b) SGLD (c) SGHMC (d) cSGLD

(e) RESGLD (f) pSGLD (g) MSGLD (h) SGNHT

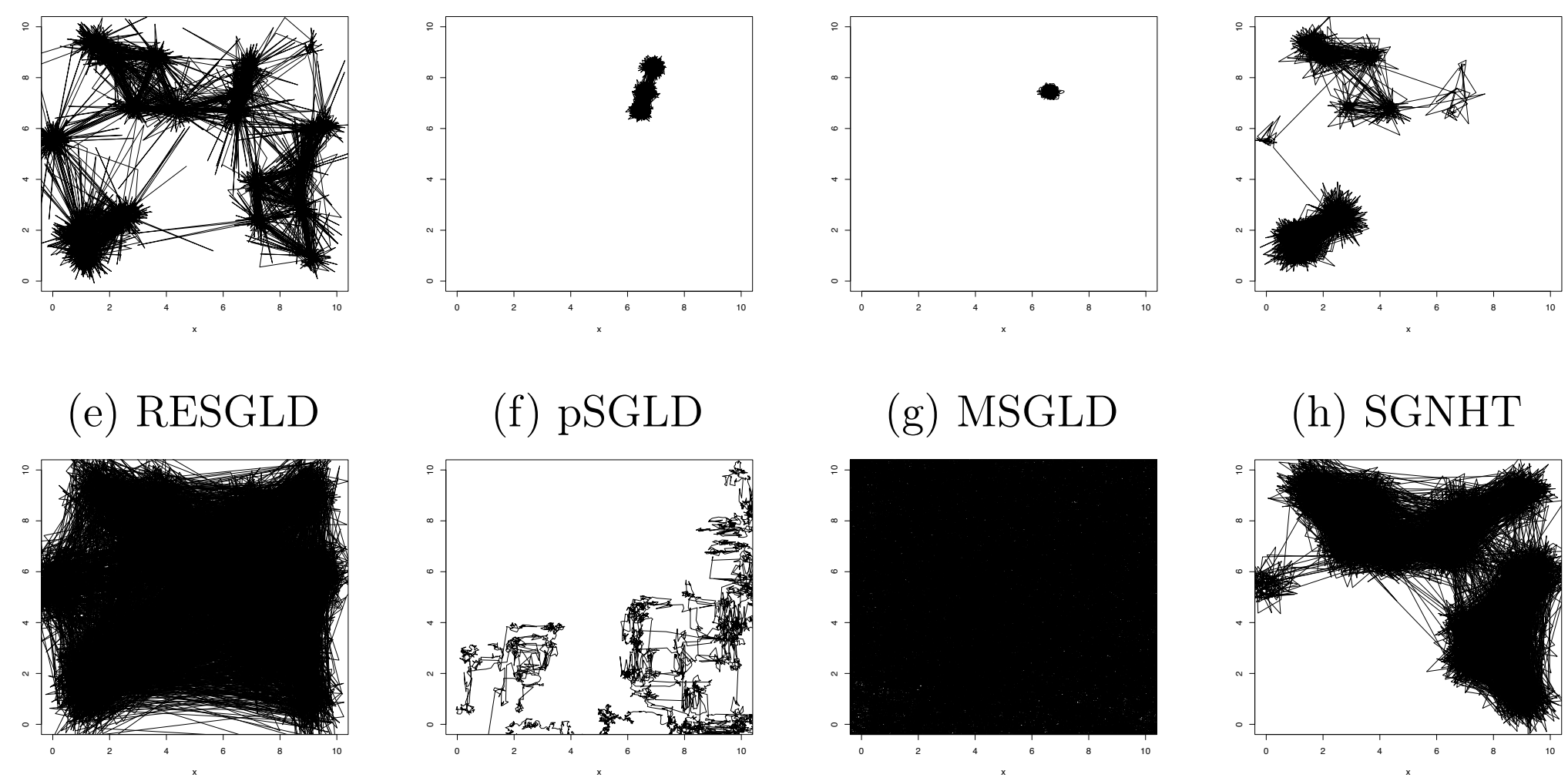

Figure 2: Sample paths produced by different algorithms with a learning rate of 0.01.

equally spaced between 1 and 7, between 1 and 10, and between 1 and 12, respectively. Under each scenario, we tried three different mutation rates: 0.4, 0.6 and 0.8, which correspond to making, respectively, 3, 2 and 1 snooker moves at each iteration. The results are summarized in Table A3.

Table A4 reports the results for the experiments conducted using ESGLD with the population size (i.e., number of chains)= 5; the learning rates being equally spaced between 0.01 and 0.03; the highest temperatures being 7, 10, and 12; and mutation rates being 0.4, 0.6, and 0.8.

Table A5 reports the results for the experiments conducted using SGLD with 10 chains, the learning rates equally spaced between 0.001 and 0.021, the highest temperatures being 7, 10, and 12, and the mutation rates being 0.5, 0.6, 0.7 and 0.8.

Table A6 reports the results for the experiments conducted using ESGLD with 10 chains, the learning rates equally spaced between 0.01 to 0.03, the highest temperatures being 7, 10, and 12, and the mutation rates being 0.5, 0.6, 0.7 and 0.8.

The four tables provide relatively consistent estimation results, despite the significantly different setups. This highlights the strength of ESGLD, which requires minimal tuning. In contrast, all other algorithms, except SGNHT, showed substantial variation in results when the setup changed, and needed retuning, particularly when the learning rate changed from 0.001 to 0.01, in order to achieve optimal performance. The reason for ESGLD's stability across different experimental setups lies in the presence of the snooker operator, which enables frequent transitions across modes, effectively mitigating the impact of changes in tuning parameters.

Table A2: The mean vector and covariance matrix estimates produced by different algorithms for the mixture distribution (10), where the algorithms were run with a learning rate of 0.01, and each estimate was calculated based on the samples simulated in 20 independent runs.

parameters	true	ESGLD	RESGLD	cSGLD	SGLD	SGHMC	pSGLD	MSGLD	SGNHT
$\mathbb{E}(X_1)$	5.403	**5.558**	5.096	5.126	4.412	5.061	4.521	4.996	5.032
SD		0.623	0.220	2.762	3.280	3.057	3.108	0.008	1.823
$\mathbb{E}(X_2)$	5.493	**5.515**	5.303	6.763	5.733	5.235	5.412	5.536	5.215
SD		0.352	0.140	2.120	3.327	3.144	3.070	0.006	1.712
$\mathrm{Var}(\mathrm{X}_1)$	10.185	9.748	**10.474**	1.862	0.049	0.018	2.423	13.925	7.323
SD		0.713	0.252	1.138	0.043	0.027	1.794	0.020	3.098
$\mathrm{Var}(\mathrm{X}_2)$	7.105	**7.061**	8.804	1.541	0.160	0.020	1.917	10.555	5.348
SD		0.635	0.151	0.730	0.179	0.031	1.525	0.012	1.609
$\mathrm{Cov}(\mathrm{X}_1, \mathrm{X2})$	-0.327	-0.727	-0.797	**-0.238**	-0.003	-0.006	-0.137	-0.601	-2.404
SD		1.388	0.463	0.852	0.075	0.025	0.852	0.016	2.070

Table A3: The mean and covariance matrix estimates produced by ESGLD with a population size of 5, the learning rates equally spaced between 0.001 and 0.021, three different highest temperatures (7, 10, and 12), and three different mutation rates (0.4, 0.6, and 0.8).

		T = 7			T = 10			T = 12		
parameters	true	0.4	0.6	0.8	0.4	0.6	0.8	0.4	0.6	0.8
$\mathbb{E}(X_1)$	5.403	5.700	5.740	5.647	5.546	5.194	5.673	5.666	5.275	5.303
SD		0.622	0.575	0.841	0.954	1.017	0.630	0.947	0.791	1.039
$\mathbb{E}(X_2)$	5.493	5.444	5.473	5.567	5.457	5.262	5.526	5.397	5.516	5.167
SD		0.496	0.562	0.514	0.726	0.718	0.399	0.581	0.496	0.508
$\mathrm{Var}(X_1)$	10.185	9.661	9.591	8.981	9.212	9.648	9.424	9.241	9.606	9.279
SD		1.575	1.369	1.235	1.405	1.439	1.258	1.507	1.304	1.212
$\mathrm{Var}(X_2)$	7.535	7.174	7.021	7.239	7.013	7.145	7.201	7.270	7.499	7.767
SD		0.734	0.594	0.767	0.653	0.925	0.645	0.686	0.948	2.020
$\mathrm{Cov}(X_1,X_2)$	-0.327	0.087	-0.315	-0.684	-0.259	0.469	-0.697	-0.238	-0.456	0.458
SD		2.133	1.955	1.716	1.884	1.746	1.524	2.048	1.764	0.895

Table A4: The mean and covariance matrix estimates produced by ESGLD with a population size of 5, the learning rates equally spaced between 0.01 and 0.03, three different highest temperatures (7, 10, and 12), and three different mutation rates (0.4, 0.6, and 0.8).

		T = 7			T = 10			T = 12		
parameters	true	0.4	0.6	0.8	0.4	0.6	0.8	0.4	0.6	0.8
$\mathbb{E}(X_1)$	5.403	5.356	5.320	5.449	5.719	5.469	5.399	5.522	5.500	5.320
SD		0.552	0.519	0.410	0.602	0.568	0.429	0.622	0.460	0.408
$\mathbb{E}(X_2)$	5.493	4.494	5.609	5.503	5.578	5.609	5.659	5.395	5.686	5.719
SD		0.399	0.371	0.261	0.473	0.406	0.307	0.513	0.208	0.204
$\text{Var}(X_1)$	10.185	10.028	9.739	9.282	9.287	9.552	9.113	9.649	9.405	9.235
SD		0.830	0.689	0.577	1.034	0.816	0.589	1.061	0.543	0.475
$\text{Var}(X_2)$	7.535	7.511	7.283	7.403	7.168	7.169	7.180	7.278	7.203	7.243
SD		0.690	0.545	0.473	0.752	0.659	0.576	0.762	0.410	0.457
$\text{Cov}(X_1, X_2)$	-0.327	-0.361	-0.973	-1.797	-1.064	-1.163	-1.762	-0.105	-1.446	2.004
SD		1.520	1.148	1.096	1.481	1.594	0.935	1.593	0.901	0.494

Table A5: The mean and covariance matrix estimates produced by ESGLD with a population size of 10, the learning rates equally spaced between 0.001 and 0.021, three different highest temperatures (7, 10, and 12), and four different mutation rates (0.5, 0.6, 0.7, and 0.8).

		T = 7				T = 10				T = 12			
parameters	true	0.5	0.6	0.7	0.8	0.5	0.6	0.7	0.8	0.5	0.6	0.7	0.8
$\mathbb{E}(X_1)$	5.403	5.873	5.468	5.577	5.533	5.886	5.830	5.910	5.474	5.590	5.951	5.487	6.069
SD		0.854	1.037	1.066	0.947	0.743	0.762	0.777	1.244	0.926	0.896	0.995	0.589
$\mathbb{E}(X_2)$	5.493	5.502	5.403	5.423	5.232	5.455	5.547	5.465	5.355	5.178	5.516	5.325	5.726
SD		0.547	0.586	0.555	0.863	0.490	0.471	0.549	0.555	0.640	0.396	0.563	0.345
$\text{Var}(X_1)$	10.185	9.450	9.436	9.302	9.762	9.553	9.322	9.261	8.798	10.135	8.863	9.581	8.968
SD		2.110	1.217	1.778	1.979	1.716	1.551	1.723	1.708	1.817	2.136	1.659	1.458
$\text{Var}(X_2)$	7.535	6.803	7.235	7.112	6.767	6.912	7.088	6.990	7.244	7.009	6.966	7.302	6.574
SD		0.831	0.914	0.736	0.989	0.944	0.761	0.930	1.003	0.776	0.793	0.852	0.618
$\text{Cov}(X_1, X_2)$	-0.327	-0.051	0.120	0.078	0.408	-0.034	-0.213	-0.241	0.040	0.619	-0.586	0.612	-1.078
SD		2.027	1.902	2.238	2.528	1.754	1.665	1.891	2.543	1.655	1.879	1.880	1.545

Table A6: The mean and covariance matrix estimates produced by ESGLD with a population size of 10, the learning rates equally spaced between 0.01 and 0.03, three different highest temperatures (7, 10, and 12), and four different mutation rates (0.5, 0.6, 0.7, and 0.8).

		T = 7				T = 10				T = 12			
parameters	true	0.5	0.6	0.7	0.8	0.5	0.6	0.7	0.8	0.5	0.6	0.7	0.8
$\mathbb{E}(X_1)$	5.403	5.397	5.483	5.457	5.641	5.521	5.668	5.558	5.827	5.765	5.778	5.738	5.529
SD		0.669	0.590	0.730	0.542	0.714	0.465	0.623	0.486	0.616	0.548	0.467	0.324
$\mathbb{E}(X_2)$	5.493	5.373	5.694	5.658	5.789	5.500	5.571	5.515	5.602	5.627	5.679	5.640	5.713
SD		0.442	0.436	0.437	0.353	0.285	0.364	0.352	0.251	0.341	0.329	0.329	0.330
$\text{Var}(X_1)$	10.185	9.778	9.648	9.500	9.050	9.665	9.592	9.748	9.084	9.363	9.289	9.536	9.398
SD		1.006	1.044	1.160	0.854	1.100	0.899	0.713	0.781	0.940	1.005	0.662	0.576
$\text{Var}(X_2)$	7.535	7.415	7.000	7.114	6.745	7.306	7.090	7.105	6.886	6.923	6.711	6.894	6.917
SD		0.741	0.720	0.692	0.665	1.127	0.515	0.635	0.580	0.661	0.632	0.420	0.630
$\text{Cov}(X_1, X_2)$	-0.327	-0.135	-0.834	-0.904	-1.976	-0.330	-0.903	-0.727	-1.601	-1.090	-1.109	-1.251	-1.529
SD		1.898	1.444	1.286	1.252	0.665	1.380	1.388	1.064	1.234	1.287	1.114	1.094

B.2 A Distribution with Long Narrow Energy Ravines

Experimental setup: For this example, the ESGLD algorithm was executed with a population of 20 Markov chains, employing an equi-spaced temperature ladder ranging from 1 to 5. The corresponding learning rates were equi-spaced from 0.001 to 0.021. The algorithm was run for 20 epochs, with the dataset randomly split into batches of size 100 at each epoch. During each iteration of each epoch, each Markov chain underwent a mutation move with a probability of 0.8 and a snooker move with the remaining probability. The selection temperature was set to 0.5. The snooker move was executed for 14 epochs, with temperature equaling to 1 and learning rate equaling to 0.001. The total CPU time for a single run was approximately 10 CPU minutes on a computer with a 2.6 GHz processor. We repeated the run 20 times for each dataset to obtain the estimates for θ_1 and θ_2.

For comparison, we applied the SGHMC, pSGLD, MSGLD, and RESGLD algorithms to the example, each working with a temperature of 1 and a learning rate of 0.001. Each algorithm was run 20 times for each dataset. We adjusted the number of epochs for each algorithm to ensure that each run took approximately 10 CPU minutes. The results were summarized in Table 2.

B.3 Towards Better Generalization of Neural Networks

Experimental Setup: For training the neural networks, we set the batch size to 500 for the datasets 'Protein', 'Electric', and 'kin8nm', 100 for the dataset 'Student', and 50 for the rest. We randomly

split the dataset into 90% for training and 10% for testing.

The ESGLD algorithm was executed with 20 chains for all ten datasets. It was run for 40 epochs for the datasets 'Boston', 'Energy', and 'kin8nm', and 100 epochs for the rest of the datasets. We replicated the run 20 times for each dataset. The snooker rate was typically set to 0.1, except for 'Energy' where the snooker rate was set to 0.3, and 'Boston' and 'Real Estate' where the snooker rates were set to 0.2. The resulting mean squared prediction error (MSPE), computed by averaging over 20 runs, and the standard deviation of the average were reported in Table 4. For comparison, SGHMC and RESGLD were applied to these datasets. For these algorithms, their numbers of epochs were adjusted appropriately to align their CPU cost with that of ESGLD.

Note that in all these simulations, the target temperature was set to 0.08 for 'Real Estate', 1.14 for 'Protein', and 0.01 for the remaining datasets. The target temperature was typically set to a value much smaller than 1 to facilitate the identification of local minimum energy models. An exception is the dataset 'Protein', where we set the target temperature to $\tau = 1.14$ due to its large sample size ($N = 45730$). To enforce the target temperature, ESGLD employed a temperature ladder with the ith chain's temperature being set to $0.01/0.65^{i-1}$. For 'Real Estate' and 'Protein', the 6th chain and 12th chain were used as the target chain, respectively. For RESGLD, the first chain ran with the target temperature and the second chain ran with 5 times the target temperature. A temperature annealing scheme with a rate of $1/1.02$ was also employed.

The learning rate was set to 5×10^{-9} for 'Boston', 2×10^{-9} for 'Qsar', 2×10^{-10} for 'Real Estate', 1×10^{-10} for 'Abalone' and 'Students', 1×10^{-11} for 'kin8nm' and 1×10^{-9} for the remaining datasets. For ESGLD, the ratio of the learning rates between ith and $i + 1$th chain was set to 0.8. For RESGLD, the higher temperature chain's learning rate was 1.6 times the lower temperature chain's learning rate. Additionally, a burn-in period of 15 epochs was implemented for 'Boston', 'Energy' and 'kin8nm', 20 epochs for 'Concrete', and 50 epochs for the remaining datasets.

References

Polyanskiy, Y. and Wu, Y. (2016), "Wasserstein Continuity of Entropy and Outer Bounds for Interference Channels," *IEEE Transactions on Information Theory*, 62, 3992–4002.

Raginsky, M., Rakhlin, A., and Telgarsky, M. (2017), "Non-convex learning via stochastic gradient langevin dynamics: a nonasymptotic analysis," in *Conference on Learning Theory*, PMLR, pp. 1674–1703.

Roberts, G. O. and Tweedie, R. L. (1996), “Exponential convergence of Langevin distributions and their discrete approximations,” *Bernoulli*, 2, 341–363.
